# Local Network Patterns in Protein-Protein Interfaces

**DOI:** 10.1371/journal.pone.0057031

**Published:** 2013-03-08

**Authors:** Qiang Luo, Rebecca Hamer, Gesine Reinert, Charlotte M. Deane

**Affiliations:** 1 Department of Management, College of Information Systems and Management, National University of Defense Technology, Changsha, Hunan, P.R. China; 2 Oxford Centre for Integrative Systems Biology, University of Oxford, Oxford, United Kingdom; 3 Department of Statistics, University of Oxford, Oxford, United Kingdom; Bioinformatics Institute, Singapore

## Abstract

Protein-protein interfaces hold the key to understanding protein-protein interactions. In this paper we investigated local interaction network patterns beyond pair-wise contact sites by considering interfaces as contact networks among residues. A contact site was defined as any residue on the surface of one protein which was in contact with a residue on the surface of another protein. We labeled the sub-graphs of these contact networks by their amino acid types. The observed distributions of these labeled sub-graphs were compared with the corresponding background distributions and the results suggested that there were preferred chemical patterns of closely packed residues at the interface. These preferred patterns point to biological constraints on physical proximity between those residues on one protein which were involved in binding to residues which were close on the interacting partner. Interaction interfaces were far from random and contain information beyond pairs and triangles. To illustrate the possible application of the local network patterns observed, we introduced a signature method, called iScore, based on these local patterns to assess interface predictions. On our data sets iScore achieved 83.6% specificity with 82% sensitivity.

## Introduction

Protein interactions are mediated by multiple physicochemical contacts at the interfaces between proteins. A considerable amount of research has focused on understanding the nature of such interactions [1]–[5].

The interfaces between proteins have been investigated in terms of the structural motifs they contain. In [6], the authors investigated the structural motifs (structural patterns that are observed more often than random patterns) in protein-protein interfaces in terms of helix, strand and coil. They found that the architectural motifs in protein cores and at protein interfaces share similar global features. Similarly, surprisingly few differences between the structural motifs in protein-peptide interfaces and those observed within monomeric proteins were found in [7]. These observations suggest that structural motifs alone are not enough to understand the unique properties of the protein-protein interfaces.

Many studies have also shown that amino acids with particular physicochemical properties tend to be in contact with complementary amino acids in the interface, for example via hydrogen bonds, electrostatic interactions, and aromatic ring stacking [8]–[11]. A contact map of a protein can be constructed by considering amino acids as nodes and the interactions between them as edges. Physicochemical properties (hydrophobicity, polarity, van der Waals volume etc.) can then be overlaid onto such contact maps. For example, by assigning weights to contact maps according to interaction properties, motifs were defined at interface by a clustering method in [12]. The authors divided a protein-protein interface into several clusters of residues from both proteins, and clusters which are structurally proximal are called neighboring clusters. The physicochemical characteristics between and within clusters had been investigated, and it was found that the structural and energetic properties as well as the evolutionary conservation of the residues in one cluster have significant effects on those properties of the residues in the same cluster but have little effects on the residues located in the neighboring clusters [13]. Many significant network motifs with 6 nodes, other than 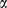-helices and 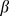-sheets, have been identified, which in turn have been used to produce a method to compare protein structures [14]. Recently, there has been rich interest in the details of different types of interactions at interfaces. In [15], the authors investigated the geometry of interactions between catalytic residues and their substrates, and found that there is no significant difference between residues involved in proton transfer and those engaged in hydrogen bonding, either in terms of distances or angles. New insights [16], [17] into the energetics at protein interfaces also suggest that the detailed computational and physical models for different types of contacts should be differentially weighted due to their different energetic contributions to complex formation, such as electrostatic interactions and hydrogen bonding interactions etc. In [18], the authors successfully identified 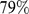 of the energetically important interactions (hot spots) by employing explicit geometry-dependent hydrogen bonding potentials.

In [19], the authors built a pair-to-pair substitution matrix for the intra-protein contact residues that are not next to one other in the amino acid sequence of the protein, and achieved relatively accurate prediction of residue-residue contacts in the protein cores from sequence information alone. The physicochemical characteristics of surface patches which were defined as a surface residue and its 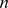 nearest structural neighbors were analyzed in [20]. The authors also applied these findings to prediction of protein-protein contact sites. They achieved 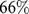 accuracy on a database of 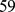 protein complexes [21]. In [22], the 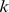-th nearest structural neighbors of the protein sites were used to form surface patches. Examining the complementarity between patches, the authors developed the SCOTCH algorithm to help protein docking methods to score candidate conformations of complexes.

In this paper we combine structural motifs, residue information and the patch idea to detect preferred patterns of interacting residues, and we illustrate our findings by providing a new scoring method for assessing interface predictions. The idea for this paper arose from our previous paper [23] where we defined an inter-protein contact site as a surface exposed residue if it is 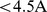 away from another surface-exposed residue on a different protein (taking all atoms into account). A contact pair consists of two residues in contact, one from each protein in a pair of binding partners, while a contact triangle has an inter-protein contact pair plus a third site with an intra-protein edge to one of the other two residues. We classified the 20 amino acids into 7 categories according to their physicochemical properties and their propensities to be in contact at protein-protein interface: Small (S,G,A,P), Hydrophobic (V,M,I,L,C), Negatively charged (D, E), Aromatic (F,Y,W), Polar (Q,T,N), Favored Positively-charged (R,H) and Disfavored Positively-charged (K). Using this reduced alphabet we counted the frequency of each type of the contact pairs and the contact triangles to establish a propensity score for contact sites. The propensity score improved the accuracy of the prediction for contact sites, but we did not investigate the details of either the patterns of the contact pairs or that of the contact triangles for the interfaces.

In this paper, we build on this work to investigate the small local network patterns termed labeled 4-tuples (pair-to-pair interactions) by considering both the structural information, the way a 4-tuple is wired, and the physicochemical properties, the amino acid composition of a 4-tuple. Four nodes can be wired as a connected graph in 6 different ways. If two contact sites from one protein and two contact sites from the other protein are connected in one of these 6 ways (for more details see Materials and Methods), these four contact sites form an inter-protein pair-to-pair interaction, called a 4-tuple. Each 4-tuple can be labeled by the amino acid types of its 4 nodes. Out of 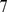 amino acid categories, we have 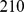 different labels for 4-tuples. In this paper, we report statistical evidence for local network patterns at interfaces, including favored and disfavored patterns of contact pairs, contact triangles, and contact 4-tuples, and show that interfaces do contain significant information beyond pairs and triangles. There are geometric and physicochemical constraints for amino acids on proteins to be able to be in contact, and ideally we would like to use these constraints to predict the interface or to assess interface predictions. While we do not know the exact constraints, they are reflected in the local pattern contents, and hence we suggest the use of the local pattern contents to predict the interface or to detect incorrect interface predictions. Exceptionality of a local pattern is judged by comparison with its surface background relative frequency, which is established under independence assumptions.

As reported in [23], the constraints imposed on each other by the residues in contact pairs and contact triangles at interfaces can significantly improve the accuracy of interface prediction in comparison to popular correlated mutation algorithms. We build a similar score as the iPatch score in [23] by using the information provided by the contact 4-tuples instead of the contact triangles. This score has advantages over previously published correlated mutation scores, but using the pattern of the contact 4-tuples did not improve the performance of iPatch for predicting the contact sites at interface (see File S1 for more details). Therefore, we conjecture that for single residue contact predictions, information from 4-tuples does not add to the information from triangles. The situation is very different for joint residue contact prediction. To illustrate the possible application of the reported local network pattern of the contact 4-tuples, we built a simple score, called iScore, based on the pattern of contact 4-tuples to select the near-native interfaces given by a docking algorithm. To filter out incorrect predictions of interfaces, a profile is established by comparing the observed local network patterns in an interface of interest with the discovered local network patterns for the interfaces in this paper. By calculating the profiles for the complex 1KU6 and its decoys reported in a docking decoy set [24], we found that the profile constructed by the labeled 4-tuples was better able to identify the correct interface in 1KU6 than either the contact pairs or the contact triangles. On a data set of 15 complexes from DOCKGROUND, with 100 decoys each and 1–10 near-native complexes each, iScore achieved 83.6% specificity with 82% sensitivity. Although we do not intend to propose an advanced scoring function for protein docking, the result given by this simple iScore also suggests that the local network patterns established in this paper capture some unique features of interfaces, and these patterns can be helpful in filtering out incorrect interface predictions. More advanced scoring function combining the local network pattern revealed in this paper with other characteristics of the interface can be expected. We conjecture that while for single residue contact predictions information from 4-tuples do not add to the information from triangles, when predicting whole protein complexes 4-tuples contain important information about co-ordinated patterns of residues from two proteins.

The background distribution depends on the database. In this paper we used three data sets, of homodimer, heterodimer, and domain-domain interfaces. For each of these data sets, we investigated the local network patterns of the interfaces in this paper, and found that the differences between these three data sets of interfaces are statistically significant. However, the profile based method gives very similar results across these three databases (see File S3 for more details). In the following, unless otherwise stated, we concentrate on domain-domain interfaces.

## Results and Discussion

### Contact sites, pairs, and triangles

Interactions between proteins are maintained by patches rather than pairs of single residues. We say that two sites at the interface are independent of each other, if the amino acid type at one site does not impose any constraint on the amino acid type at the other site. Under this assumption of independence, the relative frequency of occurrence of a pair of amino acids at the interface can be estimated by the relative frequency of one amino acid multiplied by that of the other; we call this the *background relative frequency*. However, since we know that certain amino acids are in contact with each other, in a pair of interacting residues the type of one amino acid should impose some constraints, either geometrical or physicochemical constraints, on the type of the other. Therefore, we expect to see some significant differences between the observed relative frequencies of the contact pairs or triangles and their background relative frequencies.

**Figure 1:**
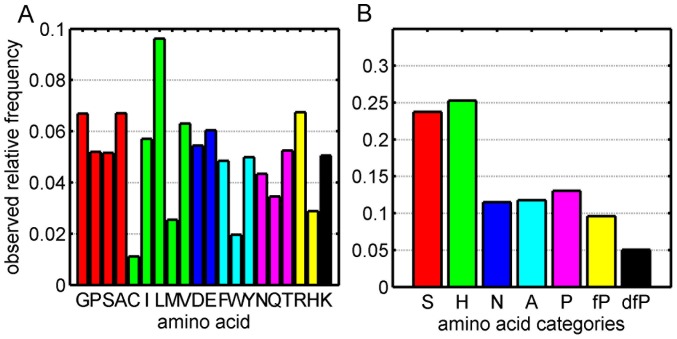
The observed relative frequencies of amino acids in interfaces. A. 20 amino acids; B. 7 amino acid categories.

As described in [23], we classified the 20 amino acids into 7 categories according to their physicochemical properties: Small (S,G,A,P), Hydrophobic (V,M,I,L,C), Negatively charged (D, E), Aromatic (F,Y,W), Polar (Q,T,N), Favored Positively-charged (R,H) and Disfavored Positively-charged (K). These categories are abbreviated by S, H, N, A, P, fP and dfP. We showed that this 7-category-grouping is useful for predicting the contact sites between proteins, which suggests that it can capture the main features of the amino acids in each category. Figure 1 shows the results of both the distribution of the 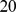 standard amino acids and the distribution of the 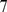 categories in the interfaces on our data set. We find that the hydrophobic and small residues are preferred at the interface. This is consistent with the observation of hydrophobic patches in interfaces [25]. We classify lysine (K) as an interface Disfavored Positively-charged residue because its observed relative frequency of occurring as an interface residue is low compared with its observed relative frequency of occurring as a surface residue. The relative propensity of lysine being in the interface against on the surface, which is calculated as the ratio of the propensity for interface over the propensity for surface [23], is 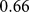, compared to the relative propensities of the other positively charged residues arginine (R) and histidine (H) which are 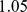 and 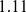 respectively. The relative frequency of cysteine (C) being found at the interface is low, but compared with its relative rarity on the protein surface, it is likely to be at the interface [23].

**Figure 2:**
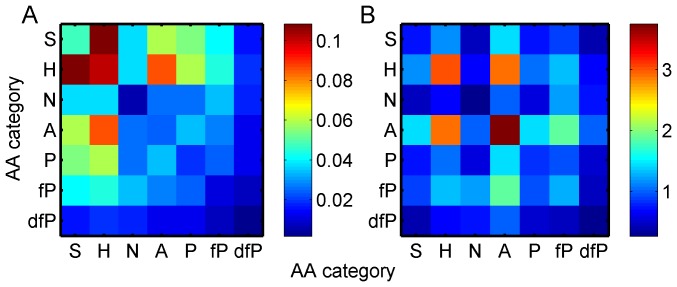
Relative frequencies of contact pairs. **A.** Observed relative frequencies of different types of contact pairs at the interface. B. Ratios between the observed relative frequencies of pair types at the interface and their background relative frequencies on the surface.

For the rest of this paper, we focus on our 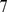 categories instead of the standard 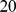 amino acids. Out of 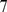 categories, we can form 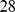 different category-category pairs and 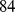 category-category-category triangles if the order of the categories does not matter. As shown in Figure 2A, some pairs of amino acid categories are found more frequently at the interface than others. For example, the most favored pairs are found among small, hydrophobic and aromatic residues. Burying the hydrophobic patches on the protein surfaces is often thought to provide the driving force for binding between proteins. Small residues as suggested by [9] can easily pack with other residues. However, the observed relative frequency also reflects the properties of protein surfaces, since the high probability for the pair, for example, of a hydrophobic residue (H) and a small residue (S) may be due to the high frequency of small residues on the protein surfaces. The under-representation of charged-charged pairs may be result from the rarity of charged residues on the surface. To help discriminate the nature of the interface from that of the surface, the ratios are calculated of the observed relative frequencies of the contact pairs in the interfaces over their background relative frequencies on the surfaces (Figure 2B). Comparing Figure 2B with Figure 2A, we see the nature of the interface when not confounded by the properties of the surface. In [9], the authors noted that the couplings of charged-charged residues are under-represented at interfaces. From our results, this under-representation is due to the particularly low ratio of the dfP-N, since dfP-N has a ratio of about 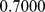 and fP-N has a ratio of 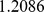. It is also interesting to see that the observed relative frequency of the pair fP-fP occurring at the interface is 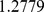 times of its surface background relative frequency. Small residues (S) do not seem to be very important for interfaces when the surface background has been excluded, and the most favored amino acid category is the aromatic (A) residue. In fact, except for Negatively charged residues (N) and Disfavored Positively-charged residues (dfP), interactions between Aromatic residues (A) and any other residue are favored at the interface, and the coupling of A–A is the most preferred. From this observation, we could infer that if we find an aromatic residue on the protein surface, it is likely to be involved in an interface.

**Figure 3:**
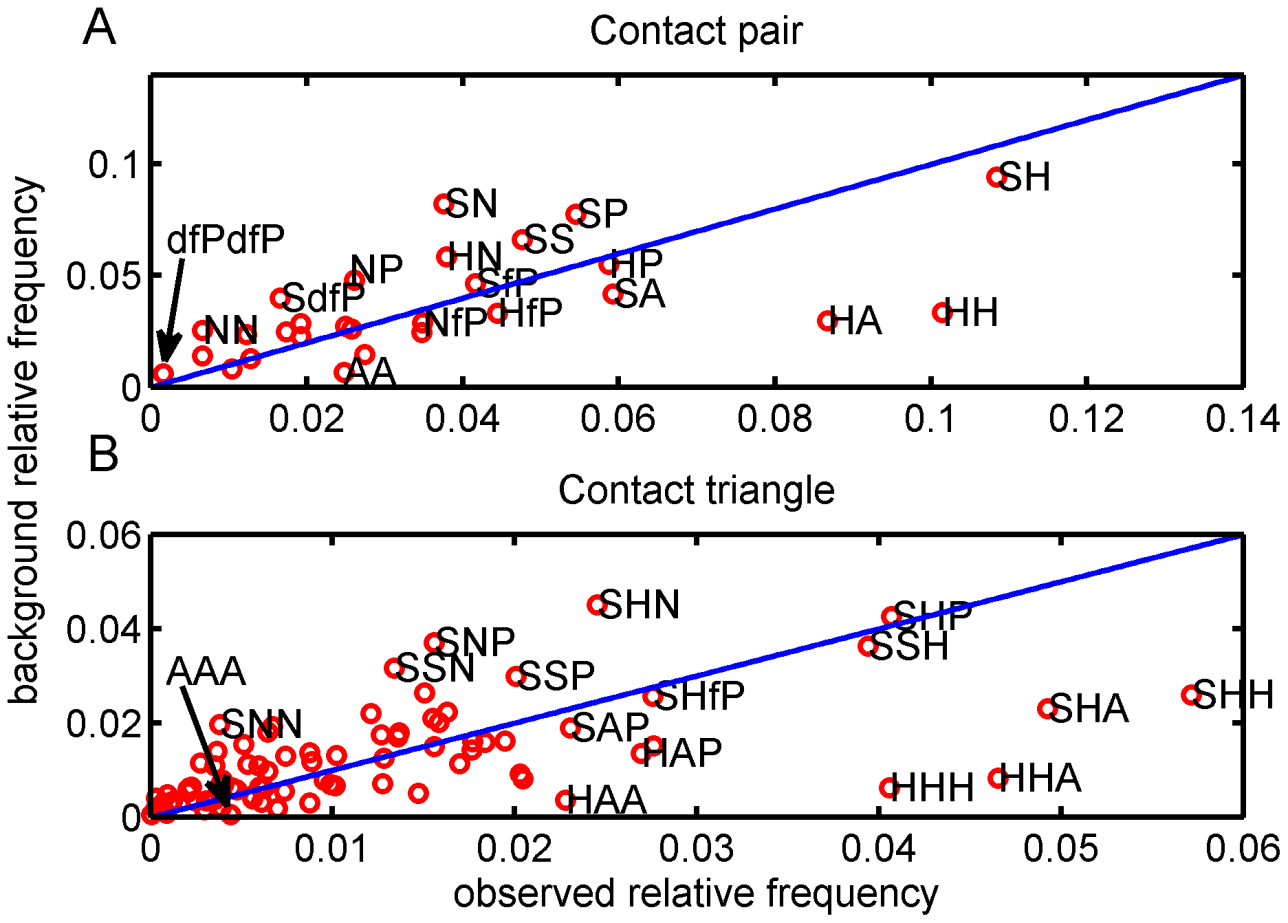
Scatter plots of observed relative frequencies and their background relative frequencies. **A.** Contact pairs; **B.** Contact triangles. The label ‘HA’ means the contact pair which consists of hydrophobic residue and aromatic residue, and the label ‘HAA’ is hydrophobic-aromatic-aromatic triangle type.

To see how significant the coupling of, or dependency between, the residues in the interface is, we compare the background relative frequencies with the observed relative frequencies of the contact pairs and triangles. The greater the difference the more significant the coupling. These two relative frequencies are plotted against each other in Figure 3. The dots off the diagonal line suggest that the interface has favored and disfavored interactions between different amino acid categories, *i.e.* if a dot is lower than the diagonal line it is favored by the interface. In contrast, if a dot is above the diagonal line it is disfavored by the interface. Instead of using the absolute distance of the dot to the diagonal line, we use the angle between the diagonal line and the vector defined by both the dot and the origin, since our interest is in the relative difference (See Methods for more details about the relationship between the angle and the ratio). As shown in Figure 3A, the observed relative frequencies of three pairs of amino acid categories, A-A (ratio  =  3.7509), H-H (ratio  =  3.0240), and H-A (ratio  =  2.9116), have the greatest divergences from the diagonal line. In Figure 3B, the greatest divergences come from the triangles, A-A-A (ratio  =  8.1842), H-H-H (ratio  =  6.6022), and H-A-A (ratio  =  6.2720).

### 4-tuples of inter-protein contact sites

**Figure 4:**
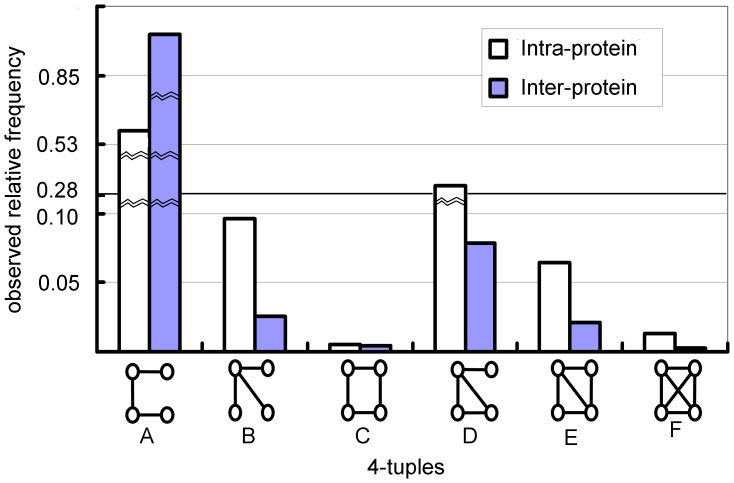
Comparison of distributions of intra-protein 4-tuples and inter-protein 4-tuples. From the left to right the 4-tuples are named from ‘A’ to ‘F’ respectively. The relative frequencies for intra-protein 4-tuples are relative to the total number of the occurrences of all types of 4-tuples in the protein interior. The relative frequencies for inter-protein 4-tuple are relative to the total number of the occurrences of all types of 4-tuples in the protein-protein interface.

There are 6 different ways to form a connected graph on 4 nodes; these can be found in Figure 4. Here we think of 4-tuples with nodes being inter-protein contact sites. We have counted the different types of 4-tuples at interfaces (two residues on one protein in contact with two residues on another protein) as well as the 4-tuples composed of intra-protein interactions (four residues in “contact” within the same protein). Figure 4 shows the relative frequencies of 4-tuples in the protein interior and at the protein-protein interface. For intra-protein interactions, we have a larger number of counts for subgraph ‘F’: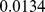, than ‘C’: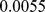; while for inter-protein interactions, it the converse is seen as the relative frequency of ‘F’ is 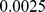 less than the relative frequency 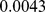 of ‘C’. This is probably due to the rigidity requirement being different between intra- and inter- protein interactions. From subgraph ‘A’ to ‘F’, the rigidity of the local network is increasing, while the flexibility is decreasing. There are many more counts of subgraph ‘A’ at protein-protein interfaces than in protein interiors, and fewer counts of subgraphs ‘D’, ‘E’, ‘F’ suggesting that the protein-protein interface is more flexible than the protein interior. This is understandable as the protein interior needs to have enough rigidity to maintain the protein structure.

**Figure 5:**
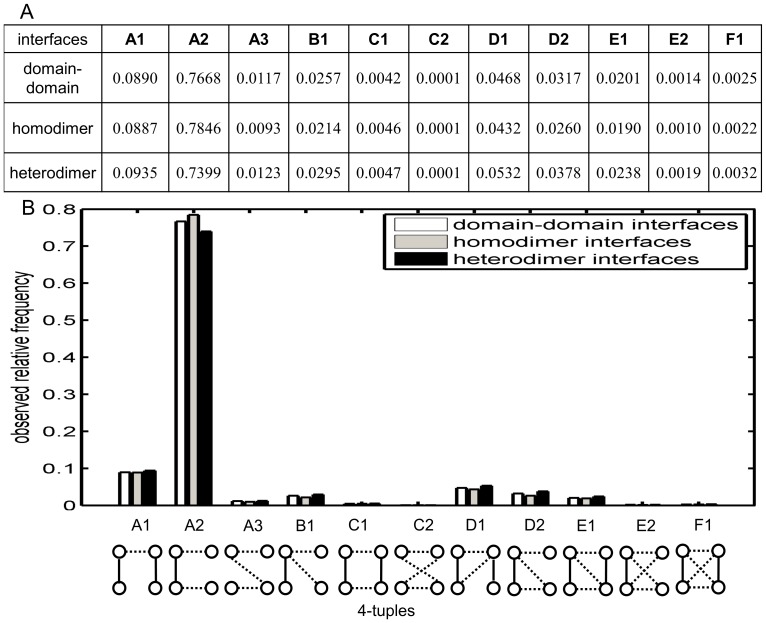
4-tuples of inter-protein contact sites. In the 4-tuples, the dotted edges stand for inter-protein contacts, while the solid edges are the intra-protein contacts. The subversion k of category X is named Xk. *e.g* the sub-version 1 of type A is named A1. A. The observed relative frequencies. B. Relative frequencies of 4-tuple subversions at interfaces.

Figure 5 shows the relative frequencies of contact 4-tuples at inter-protein interfaces in our data sets (See File S2 for the count results of labeled 4-tuples at interfaces). According to [26], the high frequency of the subgraph ‘B’ and low frequency of subgraph ‘F’ usually indicate that the structure of the underlying systems is too complicated to be described with only a few parameters. Figure 5A lists the observed relative frequencies of these 4-node-subgraphs. Compared with subversion C1, A2 requires only that two contact sites on one protein surface are also intra-protein neighbors, while C1 asks for intra-protein contacts on both proteins. Subversion A2 is the most frequent 4-node-subgraph at interface, while C1 is even less frequent than E1. The abundance of A2 and the rarity of C1 suggest that many inter-protein interactions are formed by the contacts between one patch on one protein and two patches on the other. It is interesting to see that C1 is almost as rare as F1 at interfaces; another observation is that while E1 requires more contacts than C1 does, it is more frequent than C1. This observation suggests that if the inter-protein interactions are maintained between only two patches across an interface, A1 is the most frequent contact 4-tuple; and if this interaction is tight, then it tends to be as tight as E1. Finding more occurrences of F1 than of E2 at an interface also suggests a tendency of clustering in binding.

When both intra-protein contacts are present, which is the case for A1, C1, D1, E1, F1, one would assume that the more inter-protein contacts, the less frequent the subgraph. However, this is not always the case: E1 is more frequent than C1 despite more contacts; C1 and D1 have the same number of inter-protein contacts, yet D1 is far more frequent. When only one intra-protein contact is present, as in A2, B1, D2 and E2, then D2 is more frequent than B1 despite requiring one more inter-protein contact. This indicates that there is a tendency to complete the “triangle” which is hidden in B1. These observations could be viewed as a “tendency to create triangles” at interface. When neither of the intra-protein contacts are present, as in A3 and C2, then we see that C2 is extremely rare, so that should rule out some inter-protein contacts in the prediction: if we predict C2 then we are most likely wrong. Since every site in our 4-tuples is an inter-protein contact site, subversion A2 only asks for an extra intra-protein interaction among those four sites, while A1 requires two of them. Each of those two sites in A1 with only intra-protein edge must have at least one inter-protein edge shared with some site other than those ones in this subgraph. This explains why we observed the A2 as the most frequent pattern at interface.

**Figure 6:**
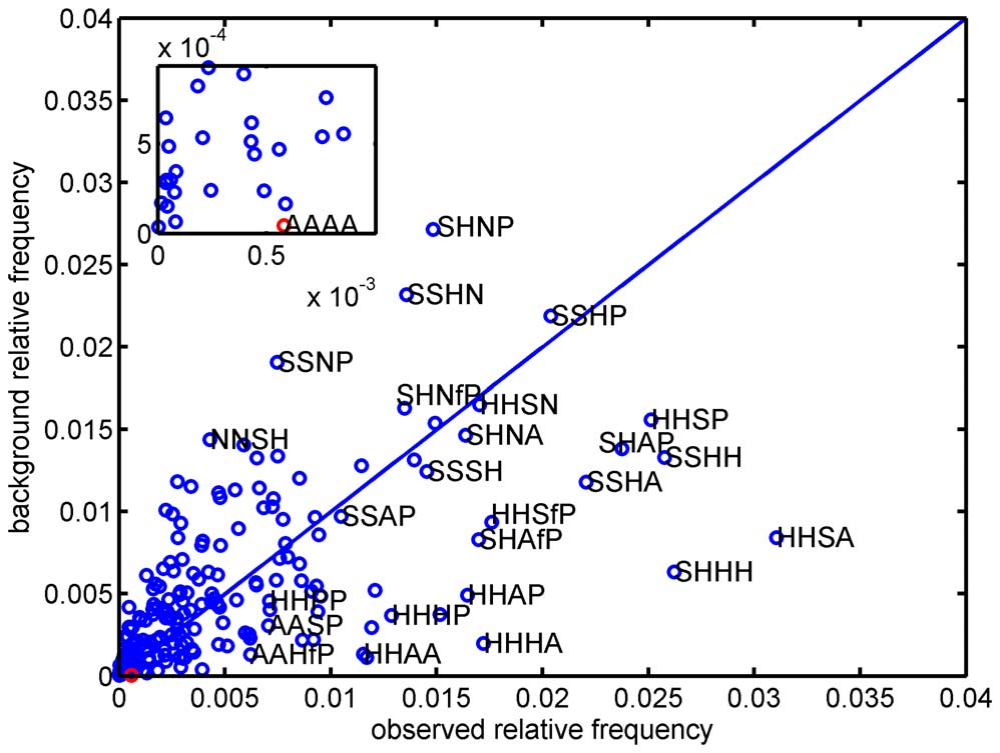
Counting of inter-protein 4-tuples in contact. Scatter plot for observed relative frequencies and their background relative frequencies for 4-tuples. The insert is an enlargement of a particular area of the graph, and the corresponding dots are marked with red circles. ‘AAAA’ is short for the aromatic-aromatic-aromatic-aromatic 4-tuple type. ‘SHNP’ stands for the small-hydrophobic-negative-polar 4-tuple type. ‘SHHH’ and ‘HHSA’ are the small-hydrophobic-hydrophobic-hydrophobic and hydrophobic-hydrophobic-small-aromatic 4-tuple types, respectively. The 4-tuples of ‘HHSA’ and ‘SHHH’ have the highest relative frequency to be contact 4-tuples, while ‘SHNP’ is expected to be the most frequent one at interface according to the background frequencies of S, H, N, and P on the surface. ‘AAAA’ is the type of contact 4-tuples with the largest divergence angle from the diagonal line.

Furthermore we considered labeled 4-node-subgraphs, with the label referring to the 7 categories. We note that there are 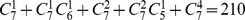 different types of labeled 4-tuples using our 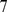 amino acid categories; here 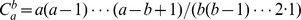. Similarly to contact pairs and contact triangles, the comparison of background relative frequencies and the observed relative frequencies for different types of 4-tuples is shown in Figure 6. We calculated the ratios of the observed relative frequencies of the labeled 4-tuples over the corresponding background relative frequencies on the surfaces. The 4-tuples of H-H-S-A and S-H-H-H have the highest relative frequency to be contact 4-tuples, while S-H-N-P is expected to be the most frequent at the interface according to the background frequencies of S, H, N, and P on the surface. Against the background of the surfaces, the most significant 4-tuple in the interfaces is A-A-A-A.

We also counted the motif of ‘1-to-k’ with 1 residue on one protein and 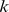 residues on the other protein in out data set. As expected, the results suggest the similar conclusion that the larger residues, aromatic residues and hydrophobic residues, tends to be the residue in contact with many residues from the other protein.

The chi-square goodness-of-fit test [27] is applied to assess whether the observed pairs, triangles, and 4-tuples can be explained by the relative frequencies of different types of amino acid categories occurring in the interfaces. We tested the null hypothesis that the observed contact triangles and contact 4-tuples can be given by the observed relative frequencies of different types of contact pairs. All the tests for contact pairs, triangles, 4-tuples reject the null hypothesis with the 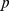-value far less than 0.0001. Therefore, there is evidence that the observed local network patterns at the interfaces cannot be explained by the relative frequency of the observed amino acid types at the interfaces. The triangles and the 4-tuples do contain significantly more information than the contact pairs, suggesting that various constraints have been introduced by the physicochemical properties of the amino acid categories to its neighborhood at interface.

### Screening the predicted interfaces by the local network patterns

**Figure 7:**
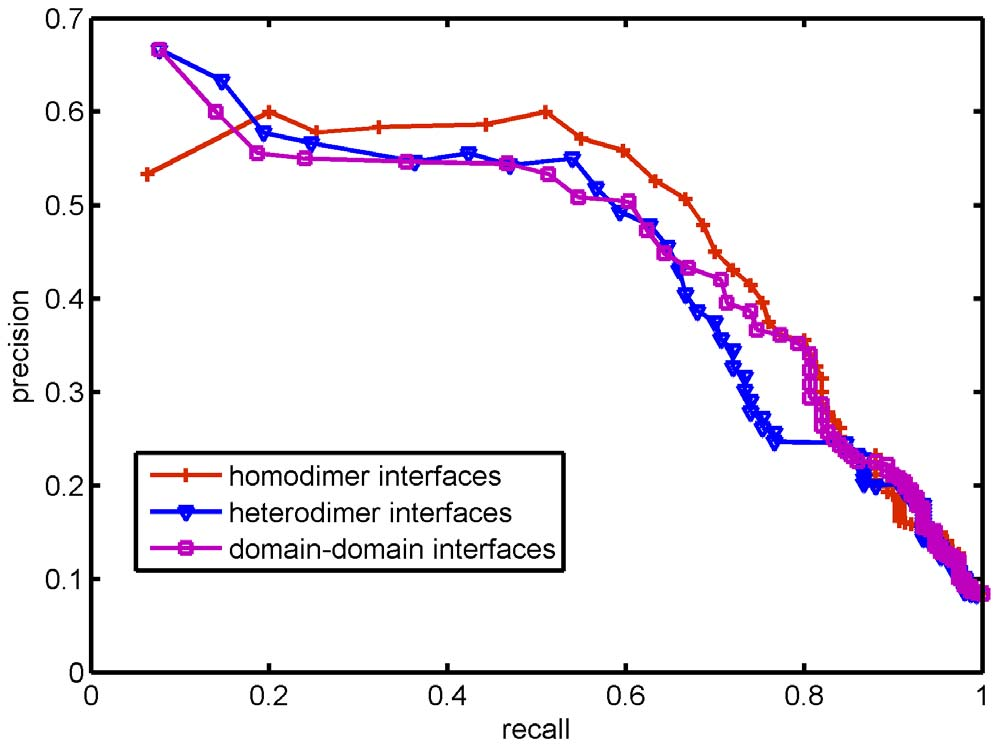
PROC for iScore based on different data sets. The precision and recall for the scores based on each data set were averaged over the results on 15 complexes from DOCKGROUND.

Local network patterns were summarized using iScore (defined in the materials and methods), and iScore was used to screen protein-protein interfaces. Among the 15 complexes in DOCKGROUND which have an interface given by only two chains and 100 decoys and 1-10 near native structures, the lowest iScore was a decoy for all 15 complexes; the highest iScore was a near-native structure for 10 of the complexes; the top 5 highest iScore's contained at least one near-native structure for 13 of the 15 complexes. The power of the method for selecting near-native structures can be measured by the average specificity and the average sensitivity for all 15 complexes. Table 1 shows that iScore achieves up to 83.6% specificity with 82% sensitivity, with similar levels across the three data sets. Figure 7 gives the average PROC curves across the 15 complexes; across the three data sets, iScore achieves around 60% precision at 20% recall.

**Table 1:** Comparison of the best performance achieved by iScore on three datasets.

Data set	Top N	Specificity	Sensitivity
Domain-domain interfaces	23	83.60%	82.00%
Homodimer interfaces	24	82.67%	82.67%
Heterodimer interfaces	28	78.87%	84.67%

The best performance was defined by selecting the top N scored structures to reach the highest possible specificity and sensitivity at the same time.

**Figure 8:**
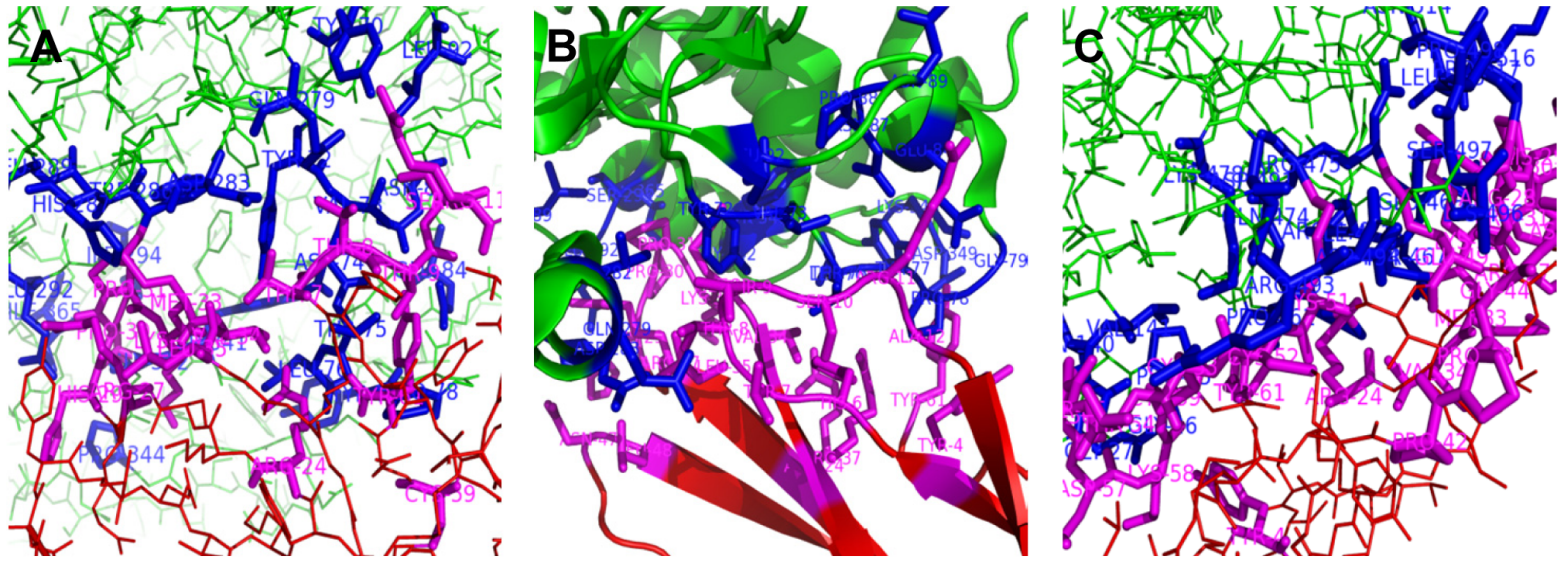
Structures of the interfaces. The structures have two chains, chain A and chain B, marked in green and red, respectively. In the interface, the contact sites on chain A are highlighted in blue, while those on chain B are in magenta. (A)r-l_51; (B)1KU6;(C)r-l_182529.

**Figure 9:**
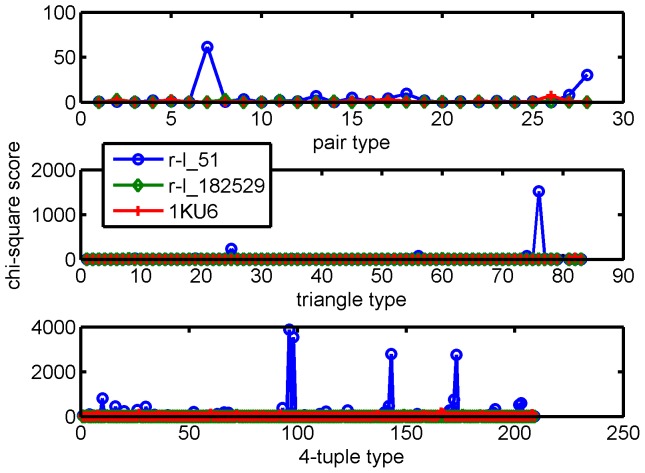
Comparing the signatures of the correct 1KU6 interface with that of decoys. The signature of a complex is the vector of chi-square scores calculated by comparing the local network patterns in the predicted interface with the profiles of those patterns revealed in this paper. The 4-tuple signature reveals most clearly that the non-near-native decoy r-l_51 deviates from the background, whereas r-l_182529 is closer to the background and has a near-native interface.

**Figure 10:**
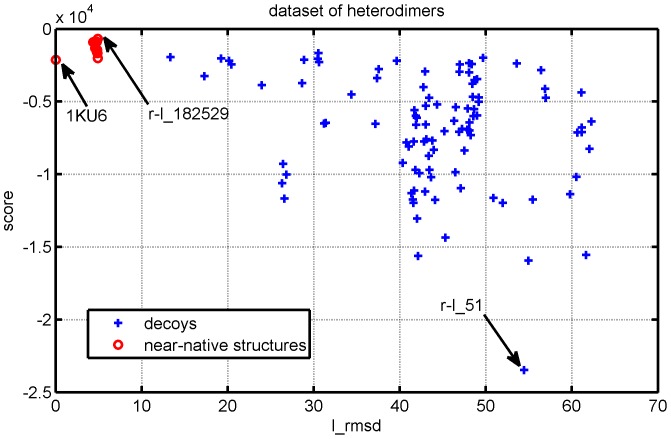
Scores against l_rmsd. Scores established by the profiles of the local network patterns given by heterodimer interfaces. l_rmsd: the RMSD of the backbone atoms of the interface residues after they have been optimally superimposed. r-1_182529 has a near-native interface and has the highest iScore, and r-1_51 is a poor decoy and has the lowest iScore.

To see how this score works, we take 1KU6 as an example. In DOCKGROUND, there are 100 decoys for 1KU6 numbered from r-l_1 to r-l_100, and 10 near-native structures named as ‘r-l_30133’, ‘r-l_30306’, ‘r-l_31538’, ‘r-l_49723’, ‘r-l_71222’, ‘r-l_81617’, ‘r-l_94327’, ‘r-l_161170’, ‘r-l_182529’ and ‘r-l_207655’. The structure with the highest iScore was a near-native structure, r-l_182529, the structure with the lowest iScore was a decoy, r-l_51, and the true interface in 1KU6 was ranked within top 17. Figure 8 shows the 3D structures of the interfaces in the complexes 1KU6, r-l_51, and r-l_182529. The RMSD of the backbone atoms of the ligand after the receptor was optimally superimposed is denoted by l_rmsd in DOCKGROUND, and the l_rmsd's of r-l_51 and r-l_182529 are 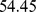 and 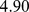, respectively. The chi-square signal was calculated as described in the Method section for the contact pairs, triangles, and 4-tuples, respectively, and the results are reported in Figure 9. The chi-square scores are calculated for 28 types of contact pairs (upper graph in Figure 9), 84 types of contact triangles (middle graph in Figure 9) and 210 types of contact 4-tuples (lower graph in Figure 9). The pair-type signature is not very informative, the triangle-type signature is somewhat informative; it is the 4-tuple signature which most clearly indicates that the decoy r-l_51 deviates from the background, whereas r-l_182529 is a near-native interface. As shown in Figure 10, the results suggest that the near-native structures as well as the real structure generally have higher iScores than the decoys. We observed that 6 out of 100 decoys exhibit a score at least as large as 1KU6. If we were thinking about the classification problem as a hypothesis test, then the probability that 1KU6 would be classified incorrectly was 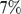. The figures of iScore against l_rmsd for all 15 complexes in the data set have been presented as Figure S7 in the File S3.

In this paper, we investigated the interactions of up to 4 residues in the interfaces between proteins from a statistical viewpoint. Considering the interfaces as networks with nodes of residues and edges of contacts, we examined labeled contact pairs, triangles, and 4-tuples. On our data set, the difference between the observed relative frequencies of those labeled subgraphs across the interfaces and the corresponding background relative frequencies gives an idea of how significant the existence of such preferred patterns is. These preferred patterns point to biological constraints on physical proximity between those residues on one protein which are involved in binding to residues which are close on the interacting partner (C2 is extremely rare). The statistical tests suggest that higher order labeled motifs have significantly more information than what can be inferred by lower order motifs. Computationally, we find that interaction interfaces are far from random and contain information beyond pairs and triangles. We exploited this fact by providing a signature method for interfaces. A chi-square score, called iScore, was calculated by comparing the profiles of the local network patterns built in this paper with those patterns in the predicted interfaces. The results suggest that the signal for the decoy established by the profile of the contact 4-tuples is stronger than that established by either the contact pairs or the contact triangles. When based on the contact 4-tuples the score achieves high specificity and sensitivity. The score demonstrates that the local network patterns studied in this paper do capture some characteristics of protein-protein interfaces. Future work will investigate alternative scoring methods.

## Materials and Methods

### Data sets

The main data sets used in this study were built as described in [23]; here is a brief review. Three data sets of 1150 two domain proteins, 583 homodimers, and 94 heterodimers were used in this paper to discover the local network patterns at interfaces. Each entry in the database has a 3-D structure with a resolution better than 2.5Å. The interface residues were identified as residues which are 4.5Å  or less away from a residue on the other protein or domain. Domain annotations were collected from SCOP [28], while the complexes were gathered by querying the PDB [29]. Sequence identity in the database is less than 70%, the change in the accessible surface area (ASA) on binding for all proteins is greater than 175Å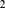, and the sequence length of each chain is more than 100. Notably, the crystal contacts are difficult to be excluded completely from the database since a crystal contact may bury as much as 800Å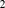 of the surface area [30]. However, in this paper, the possible crystal contacts in the protein complexes can only be a small portion of the whole data set of interfaces since we have chosen the oligomeric state of 2 in PDB for the complexes. The set of two-domain proteins comes from SCOP and will not have crystal contacts. While the count numbers of different types 4-cliques at interfaces of the combined database including all three types of interfaces and the corresponding numbers counted at the domain-domain interfaces have a correlation coefficient of 0.9988, a chi-square test rejects the hypothesis that the distributions are the same across the three databases. The domain-domain interfaces being the largest of our three data sets, we first use the data set of the domain-domain interfaces to discover the local network patterns for interfaces, and then compare the findings to that of the homodimer interfaces and heterodimer interfaces to reveal more subtle characteristics for the interfaces. In the results section, unless specified explicitly the data set mentioned in the Result section means the data set of the domain-domain interfaces.

To assess the relevance of the preferred patterns, we proposed a scoring method for selecting the near-native structures from docking decoys. We selected all complexes with the interface given by only two chains, A and B, from DOCKGROUND yielding 15 complexes, which are listed in Table 2. For each complex, the set contains the top 100 nonnear-native structures with the highest surface complementarity scores [24]. In DOCKGROUND, the near-native structure is defined as the docking solution with l_rmsd less than 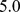, where l_rmsd is defined as the RMSD of the backbone atoms of the ligand after the receptor was optimally superimposed. The number of near-native structures or hits included in each set ranges from 1-10 is listed in Table 2.

**Table 2:** Complex list.

ID	Complex	Class.	Rec.	Chain	RMSD	Res.	Lig.	Chain	RMSD	Res.	RMSD	Hits
1	1e96_A_B	0	1mh1	A:A	0.73	1.38	1hh8	A:B	0.62	1.80	2.82	10
2	1gpw_A_B	0	1thf	D:A	3.56	1.45	1k9v	F:B	0.69	2.40	2.59	10
3	1he8_A_B	0	1e8y	A:A	2.31	2.00	2evw	X:B	1.20	1.05	4.93	1
4	1ma9_A_B	0	1kw2	A:A	0.99	2.15	2fxu	A:B	9.53	1.35	2.86	10
5	1s6v_A_B	0	2eut	A:A	0.96	1.12	1ycc	A:B	1.97	1.23	3.18	4
6	1xd3_A_B	0	1uch	A:A	2.45	1.80	1yj1	A:B	2.73	1.30	3.64	10
7	2a5t_A_B	0	1pb7	A:A	2.73	1.35	2a5s	A:B	2.31	1.70	4.95	1
8	2ckh_A_B	0	2ckg	A:A	0.82	2.45	1wm3	A:B	0.76	1.20	2.47	10
9	3fap_A_B	0	1bkf	A:A	0.63	1.60	1aue	A:B	0.69	2.33	3.67	10
10	1avw_A_B	1	2a31	A:A	0.78	1.25	1avu	A:B	0.76	2.30	2.92	10
11	1ku6_A_B	1	2c0q	A:A	4.06	2.50	1fas	A:B	0.71	1.80	4.37	10
12	1oph_A_B	1	1qlp	A:A	3.12	2.00	1hj9	A:B	2.53	0.95	1.28	10
13	1tmq_A_B	1	1jae	A:A	0.77	1.65	1b1u	A:B	1.42	2.20	2.07	10
14	2bkr_A_B	1	2bkq	A:A	2.33	2.00	1ndd	A:B	1.02	1.60	1.58	10
15	1u7f_A_B	0	1mjs	A:A	2.26	1.91	1ygs	A:B	1.29	2.10	1.19	10

Complexes selected from DOCKGROUND to demonstrate the use of the observed local network pattern at the interface.

Class.: (1) enzyme/inhitor, (0) others.

Rec.: pdb code of unbound structure of protein 1; Lig.: pdb code of unbound structure of protein 2.

Chains before colon are in unbound structure; chains after colon are in co-crystallized structure.

RMSD: C_alpha rmsd of unbound and co-crystallized structure.

Res.: crystal structure resolution.

Hits: the number of near-native solution kept in each decoy set.

### Analysis

We defined a contact pair at the interface as two surface residues within 4.5Å of each other on different proteins, *i.e* one residue from each protein. The observed relative frequency of a contact pair of amino acid categories is defined as the ratio between the counts of this pair of amino acid categories occurring as a contact pair in our data set and the total number of all 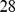 different pairs of amino acid categories occurring as contact pairs in our data set. The background relative frequency of an amino acid category was calculated as the ratio between the counts of this amino acid category occurring as an surface exposed residue in our data set and the total number of the occurrences of all 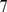 different amino acid categories locating as surface residues in our data set. The background relative frequency of a contact pair of amino acid categories was established by the product of the background relative frequencies of these two amino acid categories in this pair. Mathematically, the observed relative frequency of a contact pair of amino acid categories is denoted as
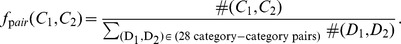



The number of occurrences, 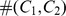, of the pair 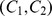 occurring as contact pair was calculated by counting the number of contact pairs of amino acids 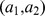 in our data set, where 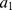 belongs to the category 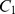 and 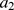 belongs to the category 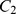. The corresponding background relative frequency of the pair, 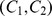, on the protein surface is given by 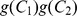, where 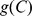 is the relative frequency of the amino acid category 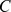 occurring on the protein surfaces, *i.e.*

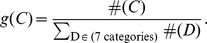



The number of occurrences 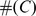 of amino acid category 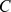 occurring on the protein surfaces was established by counting the number of surface residue 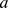 locating on the protein surfaces in our data set, where 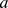 belongs to the category of 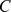. Note that for the relative frequency of a pair of amino acid categories, the ordering of the amino acid categories in a contact pair does not matter.

For three surface exposed sites, two of which come from one protein and one from the other protein, contact triangles are defined when the distance between each pair of sites in the triangle is less than 4.5Å. The observed relative frequency of a contact triangle of amino acid categories is
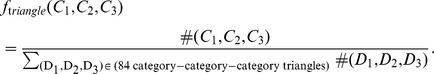



The counts, 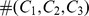, of the triangle 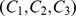 occurring as contact triangle was calculated by counting the number of contact triangle of amino acids 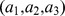 on our data set, where 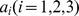 belongs to the category 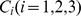 respectively. The background relative frequency of the triangle 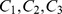 on the protein surface was calculated as 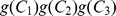, where 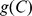 uses the same definition as described in the last paragraph. Again, the ordering of the three amino acids within a triangle does not affect the relative frequencies of the triangles. Therefore, the ratios of the contact pairs and triangles are given by
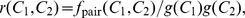



and
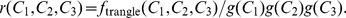



For four surface exposed sites on two proteins, two sites from each protein, 4-tuples are defined if these 4 sites are connected in one of the ways listed in Figure 4, where two sites are said to be “connected” if the distance between any two atoms in the two residues is less than 4.5Å. The inter-protein 4-tuples must include at least one inter-protein interaction, while the intra-protein 4-tuples consist of intra-protein interactions only. If we distinguish the inter-protein interactions from the intra-protein interactions in a 4-tuple, the connecting patterns of 4-tuples can be further divided into 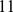 types as presented in Figure 5A. Again, since protein A binding to protein B is the same as the protein B binding to protein A, for a 4-tuple we specified that two of the sites are on protein A and two are on protein B, but we did not distinguish which two are on which protein. Therefore, the order of the amino acid categories in a labeled 4-tuple does not affect the relative frequencies of labeled 4-tuples. Mathematically, for different labeled 4-tuples, the ratios were calculated by dividing the observed relative frequency of a labeled 4-tuple occurring as a contact 4-tuple by the corresponding background relative frequency of this type of 4-tuple presenting on protein surfaces. Mathematically, for labeled 4-tuple 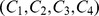, the observed relative frequency is defined as
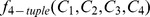


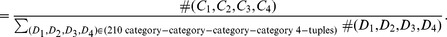



The counts, 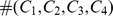, of the 4-tuple 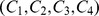 occurring as 4-tuple were calculated by counting the number of 4-tuples of amino acids 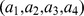 on our data set, where 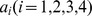 belongs to the category 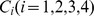 respectively. Similarly, its background relative frequency is defined as the product of the relative frequencies of amino acid categories occurring on the protein surfaces. Therefore, the ratio of a 4-tuple is given by
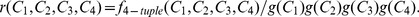
(1)


In a plot of observed relative frequency against background relative frequency, we note that the
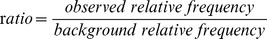
can be viewed as 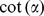, where 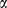 is the angle between the point (observed relative frequency; background relative frequency) and the 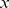-axis. Therefore, the larger the ratio, the more significant the difference.

The chi-square goodness-of-fit test [27] was applied to assess whether the observed pairs, triangles, and 4-tuples can be explained by the relative frequencies of different types of amino acid categories occurring in the interfaces as follows
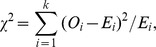
where 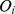 is the observed frequency for category 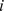 and 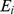 is the expected frequency for category 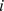. The degrees of freedom of this chi-square test is 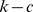, where 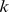 is the number of non zero cells and 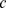 is the number of estimated parameters.

The probability of 7 types of amino acid categories occurring at interface are denoted as 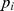 , 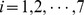. The null model for the probability 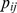 of seeing a pair 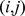 is
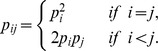



The number of estimated parameters is 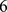, since there are 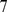 categories of amino acids, and the number of non zero cells is 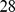 as there are 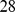 different types of contact pairs without considering the order of the amino acid types in a pair. The number of degrees of freedom for the chi-squared distribution is 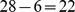. For a triangle 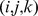, its probability given by the null model is
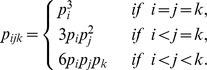



The number of estimated parameters is also 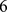, and the number of non zero cells is 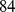. The number of degrees of freedom for the chi-squared distribution is 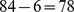. For a 4-tuple 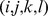, its probability under the null hypothesis of independent categories can be calculated as
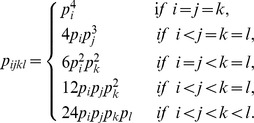



The number of non zero cells is 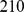, and the number of degrees of freedom for the chi-squared distribution is 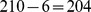.

Similarly, the test was carried out for edges to see whether the null hypothesis of contact triangles and contact 4-tuples at the interfaces can be explained by the frequency of contact pairs occurring at the interfaces can be rejected. As described above we have 28 different types of contact pairs. For a triangle 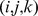, its probability 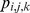 given by the null model of independent pairs is
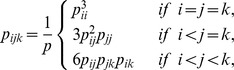
where 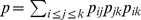. The number of estimated parameters is 27, the number of non-zero cells is 84, so the number of degrees of freedom is 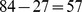.

For a 4-tuple 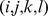, its probability given by the null model of independent pairs was calculated as
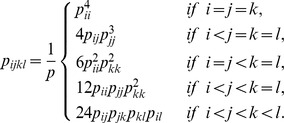



where 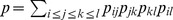. The number of degrees of freedom is 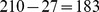.

By comparing the observed local network patters in the interface of a protein complex with the local network profiles established on our data set, a chi-square score can be calculated for this interface. Taking the coordinates of the residues in the interface of interest as the input, we built its contact map as described before; and then, we counted different types of the contact pairs, the contact triangles, and the contact 4-tuples on this contact map. For example, for the contact 4-tuple 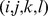, the counting results form the observation, 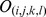 for one predicted complex, while the profiles established on our data set give the expectation, 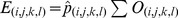, where 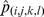 is the observed relative frequency of the contact 4-tuple, 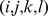, on our data set of protein-protein interfaces. Since there are 210 types of contact 4-tuples in total, the chi-square score for the 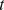-th type of the contact 4-tuples for this interface is given by
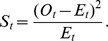



The chi-square scores for all types of contact 4-tuples establish a chi-square signature for the interface of interest, and we also carried out the chi-square goodness-of-fit between the observed pattern in the interface of interest and the profile pattern established in this paper. The protein-protein interface established by a docking algorithm was scored in terms of the above chi-square scores for all types of contact 4-tuples at the interface as follows:
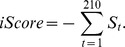



Since the near-native structures form only 7.75% of all structures in the decoy set, the PROC (Precision Recall Operating Characteristic) curve [31] will be more informative than a traditional ROC (Receiver Operating Characteristic) curve [32], especially when the score cut-off is high. Let TP stand for the number of true positives, FP for the number of false positives, TN for the number of true negatives and FN for the number of false negatives, and then
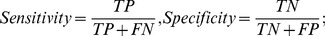


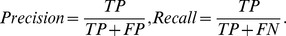



The ROC curve plots sensitivity versus specificity, while the PROC curve plots precision against recall.

## Supporting Information

File S1 Inter Protein/domain Contact Sites Predicted by Feature of 4-tuples at Interface.
(PDF)

File S2 Counts of 4-tuples in Data Sets of Domain-Domain Interfaces, Homodimer Interfaces, and Heterodimer Interfaces.
(XLS)

File S3 Comparison of Local Network Patterns Among the Data Sets of Domain-Domain Interfaces, Homodimer Interfaces, and Heterodimer Interfaces.
(PDF)
