# Supplementary material for: Local Network Patterns in Protein-Protein Interfaces
Source: PLoS One. 2013 Mar 8;8(3):e57031. doi: 10.1371/journal.pone.0057031 (PMC3592891; doi:10.1371/journal.pone.0057031)
Supplement: File S1 — Inter Protein/domain Contact Sites Predicted by Feature of 4-tuples at Interface. (PDF) [file pone.0057031.s001.pdf]

# SUPPLEMENTARY FILE I: INTER PROTEIN/DOMAIN CONTACT SITES PREDICTED BY THE FEATURE OF 4-TUPLES AT INTERFACE

QIANG LUO[1*], REBECCA HAMER[2,3], GESINE REINERT[2,3], CHARLOTTE M. DEANE[2,3]

## 1. Introduction

In the main text, we have considered the local network patterns, especially, the 4-tuples, i.e. pair-to-pair interactions as opposed to triangles or pairs. We have also showed how the different types of labeled motifs are either favored or disfavored by the interfaces through the ratios between the observed relative frequencies of these motifs and their background relative frequencies at the interfaces. The ratios are calculated from our previously built large data set of interfaces [10]. The pattern of labeled 4-tuples can also be used to predict the contact sites between domains/proteins. On a test data set which has been described in the main text, the proposed ratio score outperforms the McBASC (a correlated mutation score [15]) with exposed/buried information, the EBMcBASC score, although there is no marked improvement on using 3-tuples (TPro in our previous paper [10]).

Two case studies have been used to test our ratio score, the complex of CheAP1-CheY6 (3KYI) in *Rhodobacter sphaeroides* [1] and the complex of Spo0B-Spo0F (1F51) in *Bacillus subtilis* [17]. By pretending that the 3-D structures of the complexes are still unknown, the predictions given by our algorithms are based on the reference structures provided by

proteins from the same protein families. Comparing the predictions of the ratio scores and the real contact sites given by the complex structures, we show that the new algorithm is a promising tool for the prediction of contact sites.

## 2. Data sets

The data sets used in this study were built in our previous paper, for more details please refer to [10]. Here we will briefly review the data set. We have a database of 1150 two domain proteins and 677 protein protein complexes. The domain annotations were collected from SCOP [12] , while the complexes were gathered by querying the PDB [2]. The sequence identity in the database is less than 70%, the change in the accessible surface area (ASA) on binding must be greater than -175$Å^2$, and the sequence length of each chain must be more than 100. Each entry in our database has a 3-D structure with a resolution better than 2.5Å. In total there are 1799 proteins. This is called the propensity data set. The interface residues are identified as the residues which are at most 4.5Åaway from a residue on the other protein or domain.

Another data set is the fitting data set, which consists of 31 proteins with domain definitions annotated by literatures and 3-D structures in PDB. The sequence identity in this data set is less than 40%. The homologs of each protein are collected by running BLAST against the nr database, and the MSA is created by iteratively using MUSCLE [6] and MaxAlign [9] on the homologs of each protein.

In order to test our findings, we used a test data set in which the sequence identity to the proteins in the propensity data set and the fitting data set is less than 70%. The

proteins were selected from those proteins with two domain annotations in the latest versions of SCOP and CATH [14]. The 31 proteins in this data set have sequence identity less than 30% and 3-D structures with resolution better than 2.5Å. In order to predict the contact sites between those two domains in each protein, multiple sequence alignments (MSA) were created in the same way as for the fitting data set. Deleting columns with more than 50% gaps in the alignment resulted in 1321 contact sites remaining in this test data set.

In the chemotaxis pathway of *Rhodobacter sphaeroides*, the P1 domain of CheA transfers a phosphate group to CheY6. The MSA of the homologs of each protein were built by running Muscle, and then MaxAlign, and then Muscle again. The proteins from the CheA family and the CheY family are considered to bind to each other if they are located in the same operon in the genome. With this pairing information, concatenating the sequences of each pair leads to an MSA for the complexes in different bacteria. Two structures 1TQG [16] and 1TMY [18] both from *Thermotoga maritima* have been employed as reference structures for CheAP1 and CheY6, and the real complex structure 3KYI [1] is used to verify our predictions of the contact sites between CheAP1 and CheY6.

Specificity-determining residues in the cognate pairs of the histidine kinase (HK) and its responding regulator (RR) were predicted by the mutual information score MI and mapped onto the Spo0B-Spo0F structure [17]. We downloaded the MSA for this test case from the supplementary materials of that paper (http://www.cell.com/supplemental/S0092-8674(08)00614-4), but redo the alignment using the protocol described above.

## 3. Contact sites prediction

3.1. **Advantages in predicting of domain-domain contact sites.** We calculated a score for each site on the domain surface, which is defined by considering the structure of the domain by itself, using labeled 4-tuples in order to calculate how likely this site to be a contact site between two domains. In our previous paper, we built a package, called i-Patch, to predict the inter-domain contact sites. It uses the weighted relative propensities of contact subgraphs, the sites, pairs and triangles, which correspond to the APro, PPro, and TPro scores in i-Patch, respectively. On a test set of 31 DDI's, i-Patch propensity scores significantly outperform other correlated mutation scores, such as McBASC [15], SCA [13], ELSC [4], OMES [8], MI and its modifications [5,11] (See the paper [10] and its supplementary materials). Here, we construct a similar score, FRat, based on the ratios instead of the relative propensities of the 4-tuples. Since the 4-node-subgraph type 'A1' and 'A2' can not represent a complex interaction which consists of both multiple intra- and inter- protein interactions, at the interface, we excluded them from our calculations of ratios. FRat is designed to give high scores to those sites involved in the 4-tuples favored at the interface.

On our test data set, we have already created the MSA for each protein. Separating each protein structure in the data set into two independent domain structures, each domain will have its own surface; and we took two sites from the surface of each domain to form a 4-tuple. The background-to-surface ratios of the labeled 4-tuples were calculated as in equation (1) in Methods. Ratios were assigned to the 4-tuples in each sequence of the

MSA, and a score was given to a surface site by averaging the ratios over all possible inter-domain 4-tuples in which this site may be involved (mFRat). Again, by considering the surface patch information, we then used the weighted average of mFRat scores on the surface patch as the FRat score for each surface site. For more details see Methods. The performance of the new score was compared with that of other scores on the test data set in Figure S1. By fitting different logistic models on the fitting data set, we have also tried to combine different scores, including APro, PPro, TPro, EBMcBASC, and FRat. According to the Akaike information criterion (AIC), the logistic model (LMod) combining all these scores has the smallest AIC value, $-2.2632 \times 10^4$. The coefficients and their corresponding p-values are listed in Table S1. On the test data set, although we see some advantage of FRat over EBMcBASC, the improvement made by FRat over TPro is not significant, and thereby including FRat into the logistic model does not add significantly. In terms of the general patterns of the interactions between amino acid categories across the interface the higher order motifs bring little extra information beyond the triangles. The result also suggests that in order to improve prediction accuracy of contact sites between domains/proteins we may want to find more information about the particular contact sites between those two domains/proteins of interest rather than identifying the general patterns of the interactions between amino acid categories at the interface.

3.2. **Case studies for protein-protein contact sites.** For two proteins that are known to bind, we used the proposed FRat score to predict the inter-protein contact sites of

TABLE S1. Coefficients fitted on the fitting data set for the logistic model, LMod.

| Score | APro | PPro | TPro | FRat | EBMcBASC | Constant |
|---|---|---|---|---|---|---|
| Coefficient | 2.2949 | -2.5195 | 4.7700 | -1.1171 | 2.1679 | -5.7556 |
| p-Value | 0.0120 | 0.0000 | 0.0000 | 0.1018 | 0.0000 | 0.0000 |

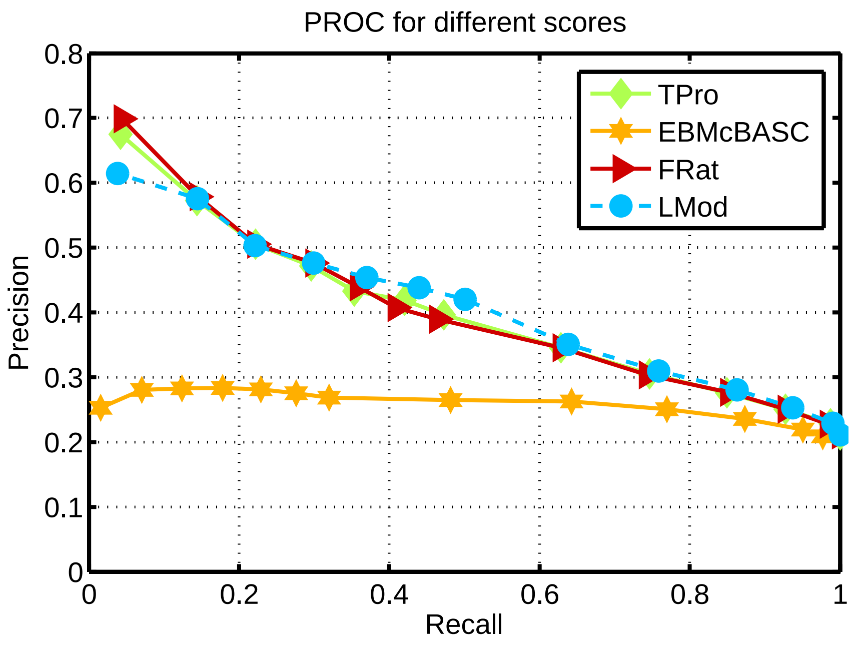


FIGURE S1. **P-ROC's for different algorithms on test data set.**

their interface. The first step is to build an MSA for each protein and at least one of the sequences in each MSA has 3-D structure (PDB file) which can be used as the reference structure to work out the surface and the surface patches for this protein. Employing some one-to-one paring information among all the homologs in these two MSA's, each sequence in one MSA was concatenated to the corresponding sequence in the other MSA to make a new long sequence. The prediction results are reported for two examples,

the CheAP1-CheY6 (3KYI) in *Rhodobacter sphaeroides*, and the Spo0B-Spo0F (1F51) in *Bacillus subtilis.*

3.2.1. *CheAP1-CheY6.* The MSA for CheA3 P1 domain and CheY6 protein from *Rhodobacter sphaeroides* were constructed as described in the data set section, together with the reference structures 1TQG (PDB record [16]) and 1TMY [18] both from *Thermotoga maritima.* Predictions were first made on the reference sequence and then mapped to the corresponding sites on the sequences of CheA3 P1 domain and CheY6 protein. Figure S2 shows that the top 40 predictions given by both FRat and TPro. There are 34 contact sites in total identified by the structure of the complex, 3KYI [1]. In our top 40 predictions given by FRat we found 12 of them. Furthermore, LMod has covered the specificity determining sites identified by [1], which are marked in blue in Figure S2.

3.2.2. *Spo0B-Spo0F.* The MSA and reference structures for this case study are described in the data set section. The prediction results given by FRat and TPro for this complex are shown in Figures S4 and S5. In fact, FRat missed 4 contact sites that successfully identified by TPro on the HK, while FRat found 1 more real contact site than TPro on the RR.

## 4. Methods

4.1. **Ratios.** For the different types of 4-tuples, the ratios were calculated by dividing the observed relative frequency of a type of 4-tuple occurring as a contact 4-tuple by the corresponding background relative frequency of this type of 4-tuple on a protein surface.

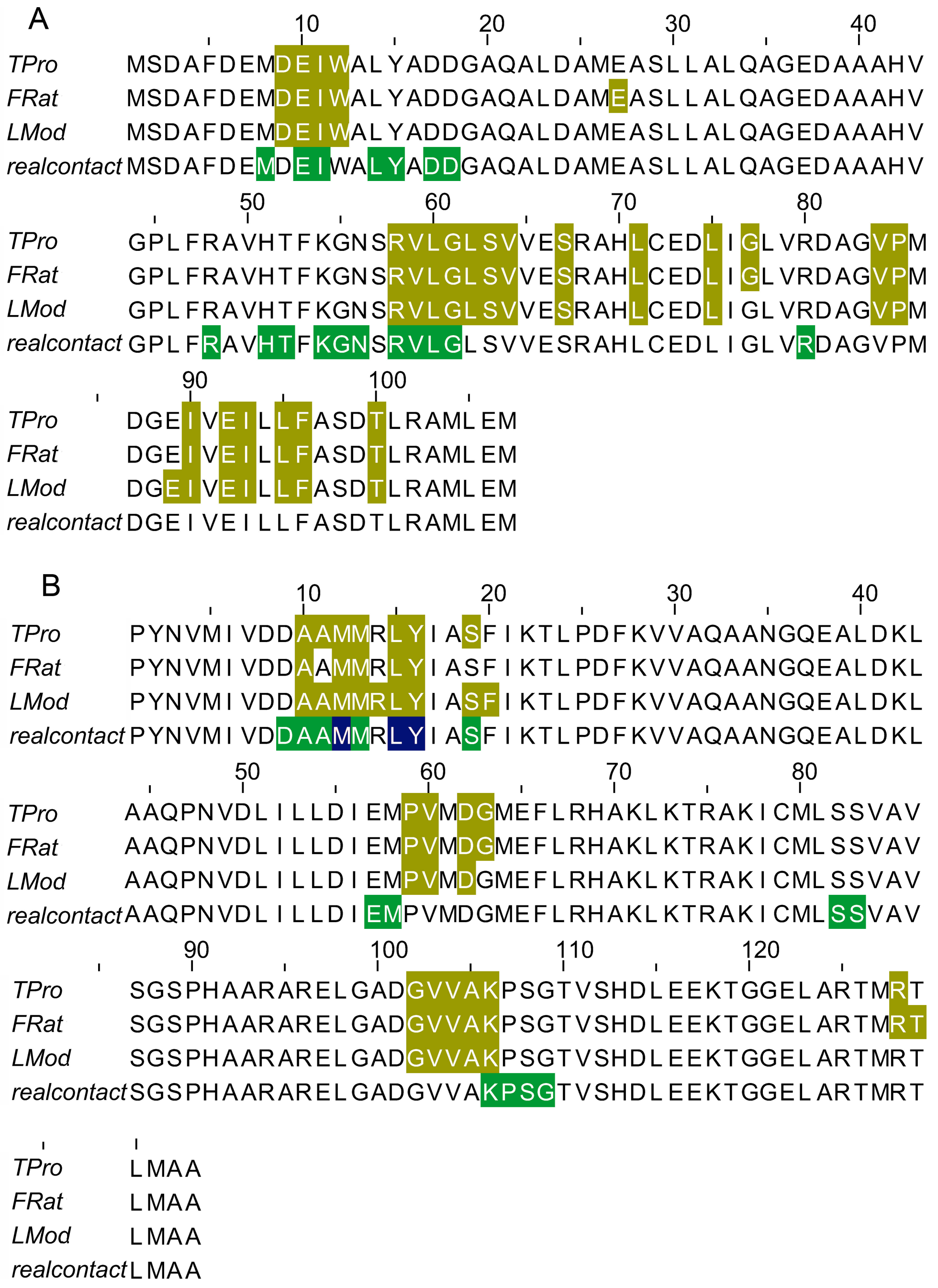


FIGURE S2. **Predictions given by different scores for CheA3P1-CheY6.** A. Predictions on CheA P1 domain. B. Predictions on CheY6 protein. Both sequences are from *Rhodobacter sphaeroides*. The top 40 predictions given by TPro, FRat, and LMod are marked with brown in the first three lines, respectively. The real inter-protein contact sites are highlighted in green on the last line, together with the specificity determining sites (which are all contact sites), according to the results reported in [1], are highlighted in blue.

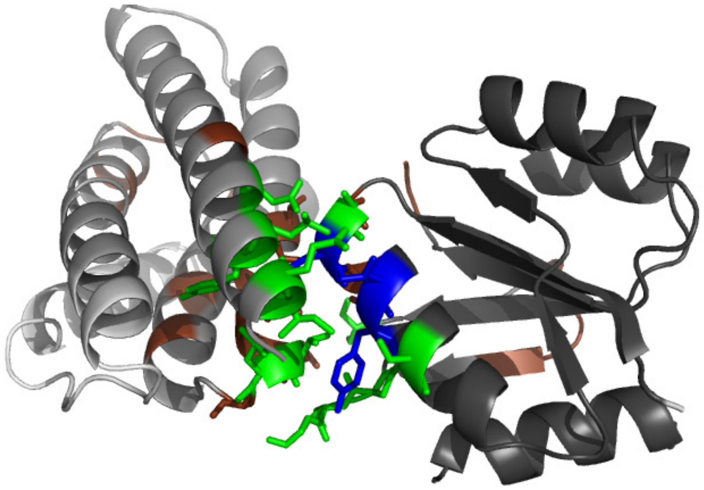

Figure S3. **Predictions on complex 3KYI.** The real contact sites are coloured in green, the predictions are shown in chocolate, the specificity determining sites on CheY6 protein [1] are highlighted in blue.

Mathematically, for all $C_1, C_2, C_3, C_4 \in \Omega_{\text{category}}$, let the observed relative frequency on the DDI and PPI data sets be $f_{4-tuple}(C_1, C_2, C_3, C_4)$ and its background relative frequency be defined as the product of the relative frequencies of amino acid categories occurring on the surface. The ratio of a contact 4-tuple is given by

$$p(C_1, C_2, C_3, C_4) = f_{4-tuple}(C_1, C_2, C_3, C_4)/g(C_1)g(C_2)g(C_3)g(C_4), \tag{1}$$

where $g(C)$ means the relative frequency of amino acid category $C$ found to be exposed on protein surfaces.

4.2. **FRat.** The 4-tuple ratio of four amino acids at sites $i_t$, $i_s$, $p$, and $q$ on the $j$th sequence in an MSA, $(a_{ji_t}, a_{ji_s}, a_{jp}, a_{jq})$, two of which come from the surface of protein

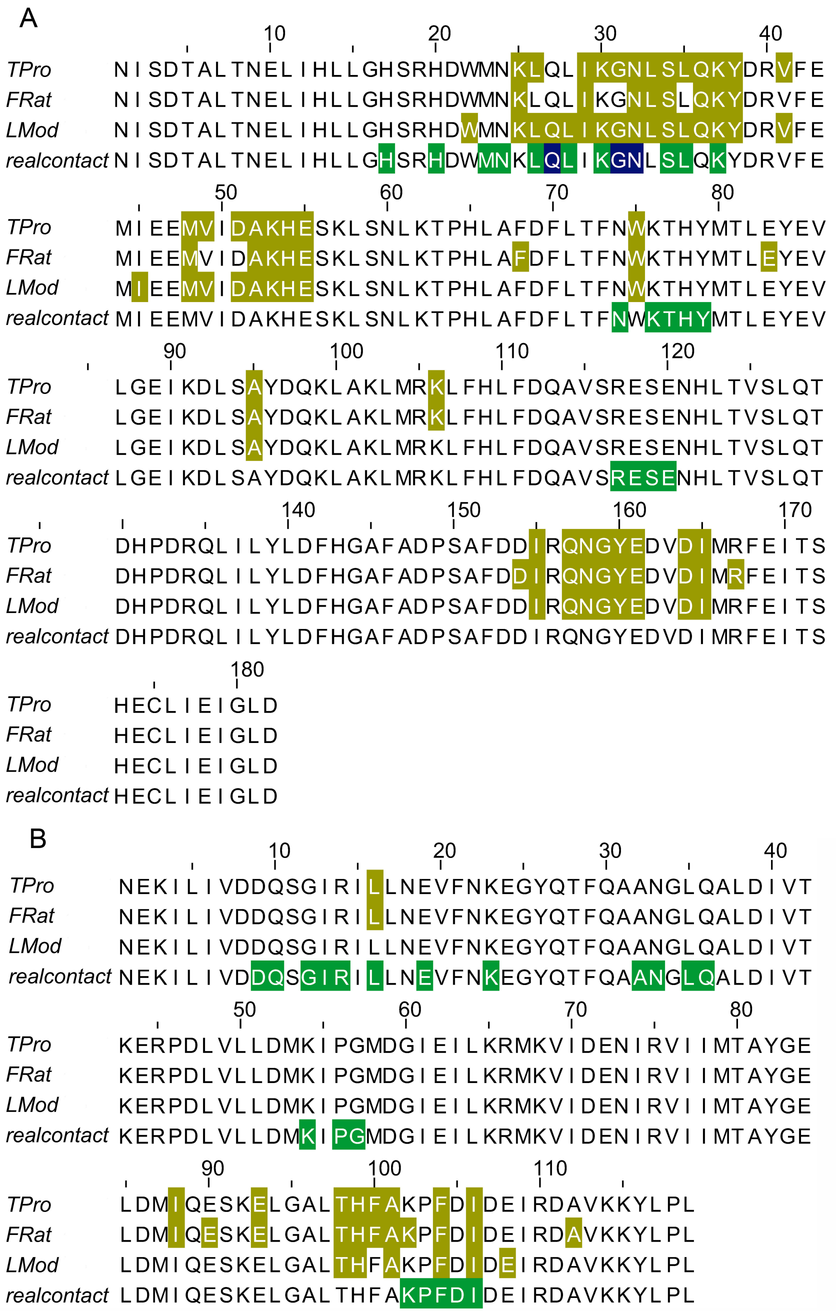


FIGURE S4. **Predictions given by different scores for the complex of Spo0B and Spo0F.** Both proteins come from *Bacillus subtilis*. A. The predictions on Spo0B; B. The predictions on Spo0F. The top 40 predictions given by TPro, FRat, and LMod are marked with brown in the first three lines, respectively. The real inter-protein contact sites are highlighted in green on the last line, together with the specificity determining sites (which are all contact sites), according to the results reported in [17], are highlighted in blue.

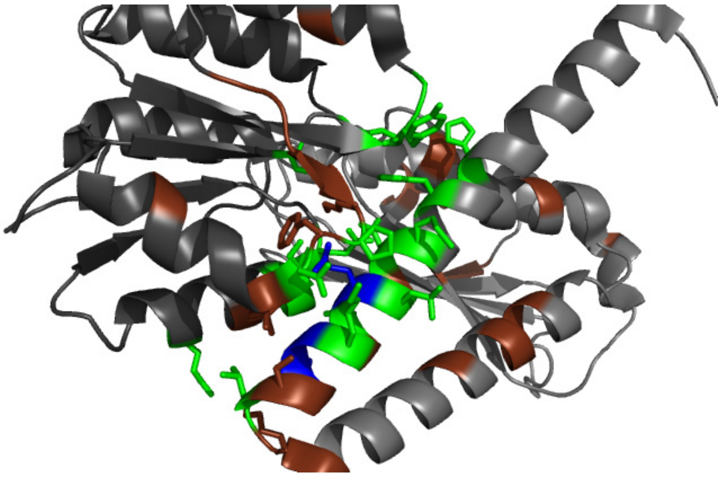

FIGURE S5. **Predictions on complex 1F51.** The real contact sites are coloured in green, the predictions are shown in chocolate, the specificity determining sites on HK [17] are highlighted in blue.

A and the other two from the surface of protein B, is given by 4-tuple ratio of the corresponding four categories, $(C_{ji_t}, C_{ji_s}, C_{jp}, C_{jq})$ as (1), *i.e.*, $p(C_{ji_t}, C_{ji_s}, C_{jp}, C_{jq})$.

For site $i$ on the surface of protein A, the set of its surface neighbours was defined as

$$\Pi(i) = \{i_k \text{ is surface exposed on the same protein: } d_{ii_k} < 4.5\text{Å}\}, \quad (2)$$

where $d_{ii_k}$ is the distance between sites $i$ and $i_k$ calculated as the smallest atom-atom distance given by the 3-D coordinates of the corresponding residues in the PDB file. Furthermore, we defined the second order surface patch as

(3)

$$\Pi^2(i) = \{i_s \text{ is surface exposed on the same protein: } \exists i_t \in \Pi(i), \text{ such that } d_{i_s i_t} < 4.5\text{Å}\}.$$

For site $i$ on the surface of protein A and site $p$ on the surface of protein B, the 4-tuple ratio calculated as (1) were assigned to a pair of neighbours of site $i$, $i_t$, and $i_s \in \Pi^2(i)$

together with another pair of neighbours of site $p$, $p_u$ and $p_v \in \Pi^2(i)$ on protein B as follows

$$S^F_{i,p} = \frac{1}{M - |G(i_t, i_s, p_u, p_v)|} \sum_{j=1}^{M-|G(i_t,i_s,p_u,p_v)|} p(C_{ji_t}, C_{ji_s}, C_{jp_u}, C_{jp_v}), \tag{4}$$

where $M$ is the number of sequences in the MSA, and $G(i_t, i_s, p_u, p_v)$ is the indexes of the sequences that have at least one gap in these four sites $i_t, i_s, p_u$, and $p_v$.

Averaging $S^F_{i,p}$ over the surface of protein B for site $i$ gives the mFRat score

$$S^{mFRat}_i = \frac{1}{|\{p \text{ exposed on protein B }\}|} \sum_{p=1}^{|\{p \text{ exposed on protein B }\}|} S^F_{i,p}. \tag{5}$$

For each surface-exposed site $i$ on protein A, the 4-tuple propensity (FRat) score was then calculated as

$$S^{FRat}_i = \frac{1}{|\Pi(i)|} \sum_{i_t \in \Pi(i)} w^{\text{intra}}_{ii_k} S^{mFRat}_{i_k}, \tag{6}$$

where $w^{\text{intra}}_{ii_k}$ is the intra protein weight [10] which profiles the preference of amino acids as the surface neighbour of the contact site $i$.

4.3. **P-ROC.** Since there tend to be fewer contact sites than non-contact sits on the protein surface, the P-ROC (Precision Recall Operating Characteristic) curve [3] may be more informative than the traditional ROC (Receiver Operating Characteristic) curve [7], especially when the score cut-off is high. Precision and recall are defined as follows:

$$\text{precision} = \frac{TP}{TP + FP}, \text{ and recall} = \frac{TP}{TP + FN}, \tag{7}$$

where $TP$ is the true positive, $FP$ is the false positive, $TN$ is the true negative and $FN$ is the false negative.

## 5. Discussion

In this supplementary material, to show that the labeled subgraphs capture some characteristics of protein-protein interface, we proposed FRat score, which uses the information of the labeled 4-tuples at the interface to predict the contact sites between domains/proteins. On a test data set, FRat outperforms the EBMcBASC score, one of the best correlated mutation scores in terms of contact sites prediction. FRat has also been used to make predictions of the contact sites for complexes in the signaling pathways of bacteria. Comparison with published experimental results shows that FRat can predict a good proportion of the inter-protein contact sites and cover some of the experimentally identified specificity sites. FRat does not predict the specificity determining sites, but it does offer a narrowing of search space by focusing on those sites within or around the predictions. Especially, the predictions given by the combination score, LMod, have covered those specificity sites identified by the latest experiments for both case studies.

Finally, we would like to suggest some future directions for this work. The FRat score does have advantages over the previously published correlated mutation scores since the multiple interactions play a key role in interfaces. However, there is no significant improvement achieved by using 4-tuples compared to use of labeled triangles to give the predictions, suggesting that the general pattern of higher order contact motifs may not improve the accuracy of prediction of contact sites significantly, and that instead we need

to use information about the particular coupling between the pair of proteins of interest beyond the general pattern of contacts.

## References


1. C. H. Bell, S. L. Porter, A. Strawson, D. I. Stuart, and J. P. Armitage, *Using structural information to change the phosphotransfer specificity of a two-component chemotaxis signalling complex.*, PLoS biology **8** (2010), no. 2, e1000306+.
2. H. M. Berman, J. Westbrook, Z. Feng, G. Gilliland, T. N. Bhat, H. Weissig, I. N. Shindyalov, and P. E. Bourne, *The protein data bank*, Nucleic Acids Res **28** (2000), no. 1, 235–42.
3. M. Buckland and F. Gey, *The relationship between recall and precision*, Journal of the American Society for Information Science **45** (1994), no. 1, 12–19.
4. J. Dekker, A. Fodor, R. Aldrich, and G. Yellen, *A perturbation-based method for calculating explicit likelihood of evolutionary co-variance in multiple sequence alignments*, Bioinformatics **20** (2004), no. 10, 1565–1572.
5. S. D. Dunn, L. M. Wahl, and G. B. Gloor, *Mutual information without the influence of phylogeny or entropy dramatically improves residue contact prediction*, Bioinformatics **24** (2008), no. 3, 333–340.
6. R. C. Edgar, *Muscle: multiple sequence alignment with high accuracy and high throughput*, Nucleic Acids Res **32** (2004), no. 5, 1792–7.
7. T. Fawcett, *An introduction to roc analysis*, Pattern Recogn. Lett. **27** (2006), no. 8, 861–874.
8. A. A. Fodor and R. W. Aldrich, *Influence of conservation on calculations of amino acid covariance in multiple sequence alignments*, Proteins **56** (2004), no. 2, 211–21.
9. R. Gouveia-Oliveira, P. W. Sackett, and A. G. Pedersen, *Maxalign: maximizing usable data in an alignment*, BMC Bioinformatics **8** (2007), 312.
10. R. Hamer, Q. Luo, J. P. Armitage, G. Reinert, and C. M. Deane, *i-patch: Interprotein contact prediction using local network information*, Proteins **78** (2010), no. 13, 2781–2797.

11. L. C. Martin, G. B. Gloor, S. D. Dunn, and L. M. Wahl, *Using information theory to search for co-evolving residues in proteins*, Bioinformatics **21** (2005), no. 22, 4116–24.

12. A. G. Murzin, S. E. Brenner, T. Hubbard, and C. Chothia, *Scop: a structural classification of proteins database for the investigation of sequences and structures*, J Mol Biol **247** (1995), no. 4, 536–40.

13. H. Najeeb, R. Olivier, L. Stanislas, and R. Ranganathan, *Protein sectors: Evolutionary units of three-dimensional structure*, Cell **138** (2009), 774–786.

14. C. A. Orengo, A. D. Michie, S. Jones, D. T. Jones, M. B. Swindells, and J. M. Thornton, *Cath–a hierarchic classification of protein domain structures*, Structure **5** (1997), no. 8, 1093–108.

15. F. Pazos, M. Helmer-Citterich, G. Ausiello, and A. Valencia, *Correlated mutations contain information about protein-protein interaction*, J Mol Biol **271** (1997), no. 4, 511–523.

16. C. M. Quezada, C. Gradinaru, M. I. Simon, A. M. Bilwes, and B. R. Crane, *Helical shifts generate two distinct conformers in the atomic resolution structure of the chea phosphotransferase domain from thermotoga maritima.*, J Mol Biol **341** (2004), no. 5, 1283–1294.

17. J. M. Skerker, B. S. Perchuk, A. Siryaporn, E. A. Lubin, O. Ashenberg, M. Goulian, and M. T. Laub, *Rewiring the specificity of two-component signal transduction systems*, Cell **133** (2008), no. 6, 1043–1054.

18. K. C. Usher, A. F. de la Cruz, F. W. Dahlquist, R. V. Swanson, M. I. Simon, and S. J. Remington, *Crystal structures of chey from thermotoga maritima do not support conventional explanations for the structural basis of enhanced thermostability.*, Protein science : a publication of the Protein Society **7** (1998), no. 2, 403–412.

**1 Department of Management, College of Information Systems and Management, National University of Defense Technology, Changsha, Hunan, P.R. China**

**2 Oxford Centre for Integrative Systems Biology, University of Oxford, Oxford, UK**

**3 Department of Statistics, University of Oxford, Oxford, UK**

*E-mail address*: mrqiangluo@gmail.com
