# Supplementary material for: Local Network Patterns in Protein-Protein Interfaces
Source: PLoS One. 2013 Mar 8;8(3):e57031. doi: 10.1371/journal.pone.0057031 (PMC3592891; doi:10.1371/journal.pone.0057031)
Supplement: File S3 — Comparison of Local Network Patterns Among the Data Sets of Domain-Domain Interfaces, Homodimer Interfaces, and Heterodimer Interfaces. (PDF) [file pone.0057031.s003.pdf]

Supplementary File III：Comparison of Local Network Patterns Among the Data Sets of Domain-Domain Interfaces, Homodimer Interfaces, and Heterodimer Interfaces

Qiang Luo[1;*], Rebecca Hamer[2;3], Gesine Reinert[2;3], Charlotte M. Deane[2;3]

1 Department of Management, College of Information Systems and Management,

National University of Defense Technology, Changsha, Hunan, P.R. China

2 Oxford Centre for Integrative Systems Biology, University of Oxford, Oxford, UK

3 Department of Statistics, University of Oxford, Oxford, UK

*** E-mail: mrqiangluo@gmail.com**

As described in the main text, we have three data sets, including the domain-domain interfaces (1150 proteins), the homodimer interfaces (583 complexes), and the heterodimer interfaces (94 complexes). In this supplementary file, all the statistics in the paper have been calculated separately for each data set.

In each section, we take the observed relative frequency of the labeled motifs at interfaces in domain-domain interfaces as the background distribution to calculate the expectations; and compare the observed relative frequency of the labeled motifs at interfaces in the homodimers and the heterodimers to the expectation by the chi-square goodness-of-fit test, which has been described in the main text. Similarly, we also compare the observed relative frequencies in the homodimers with that in the heterodimers by the same statistical test.

Contact site

With the counting numbers of different amino acid category, the correlation coefficients between any two of these three data sets are established as 0.9926 between the domain-domain interfaces and the homodimer interfaces, 0.9745 between the domain-domain interfaces and the heterodimer interfaces, and 0.9653 between the homodimer interfaces and the heterodimer interfaces. As shown in Figure S1, the observed relative frequencies of the amino acid categories are well correlated among three data sets. However, if we look into more detail, the interfaces of heterodimers have more Polar residues and Aromatic residues than the domain-domain interfaces and the homodimer interfaces, while less Small residues and Hydrophobic residues. The pairwise chi-square statistics reject the null hypothesis that any two of the data sets have the same distribution of the amino acid categories. To see the subtle contributions of each amino acid category for the statistical tests, Table S1 lists the contribution of each type of amino acid in the chi-square goodness-of-fit test for the observed samples based on different background populations.

Table S1. The chi-square contributions for different amino acid categories in the pariwise statistical tests. The observed samples is listed with its background population in the brackets.

AA	S	H	N	A	P	fP	dfP
Homo (DDI)	50.2385	0.9490	26.3625	17.2028	1.6607	30.0543	4.6060
Hetero (DDI)	13.5078	24.9604	9.2774	6.4112	23.2325	0.0165	9.6882
Hetero (Homo)	1.2471	28.3709	1.2470	17.5671	18.5445	4.1925	5.1434

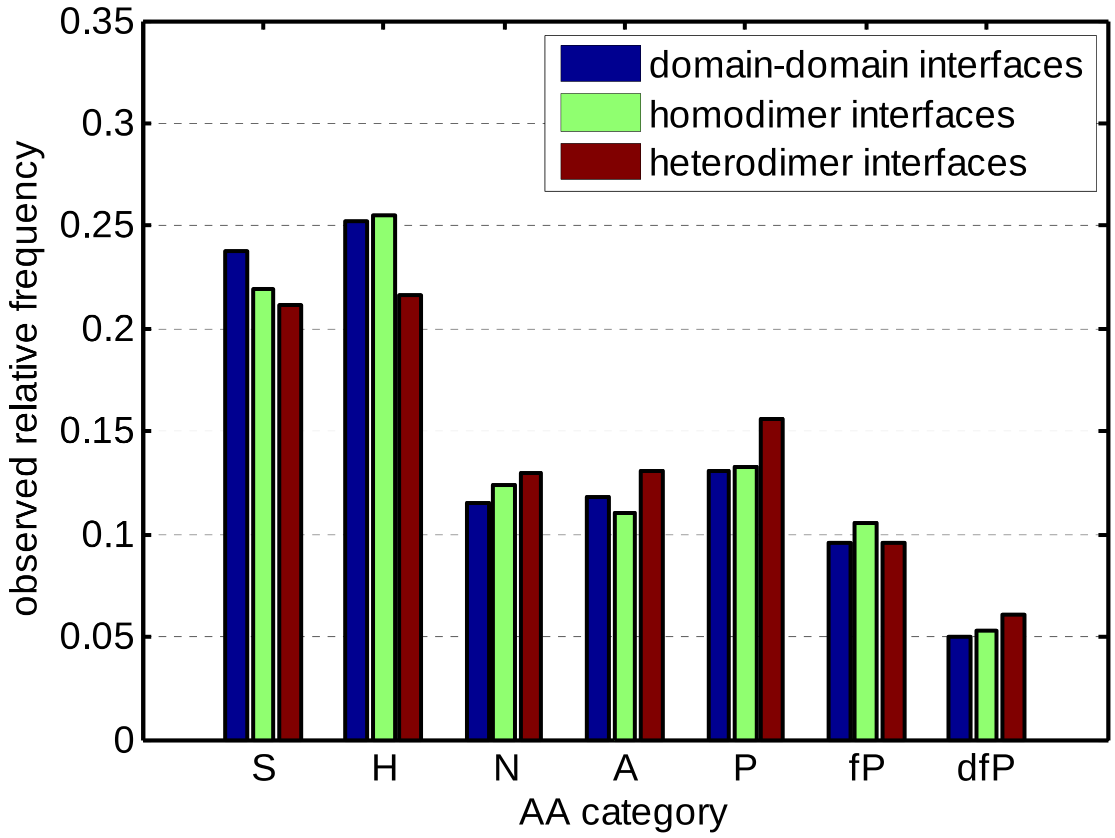

Figure S1. Comparison of the observed relative frequencies in different data sets of the interfaces.

Contact pair

In Figure S2, the observed relative frequencies of the contact pairs in three data sets are presented in the left column, and the ratios between the observed relative frequencies of the contact pairs and their corresponding background relative frequencies on the surfaces are shown in the right column. From these results we can see that the pairs of S-S and S-H are frequent at interface due to their abundance on the surface. Besides, we can also tell that the A-A, A-H and H-H are favored by the interfaces, while the dfP-dfP and N-N are disfavored by the interfaces.

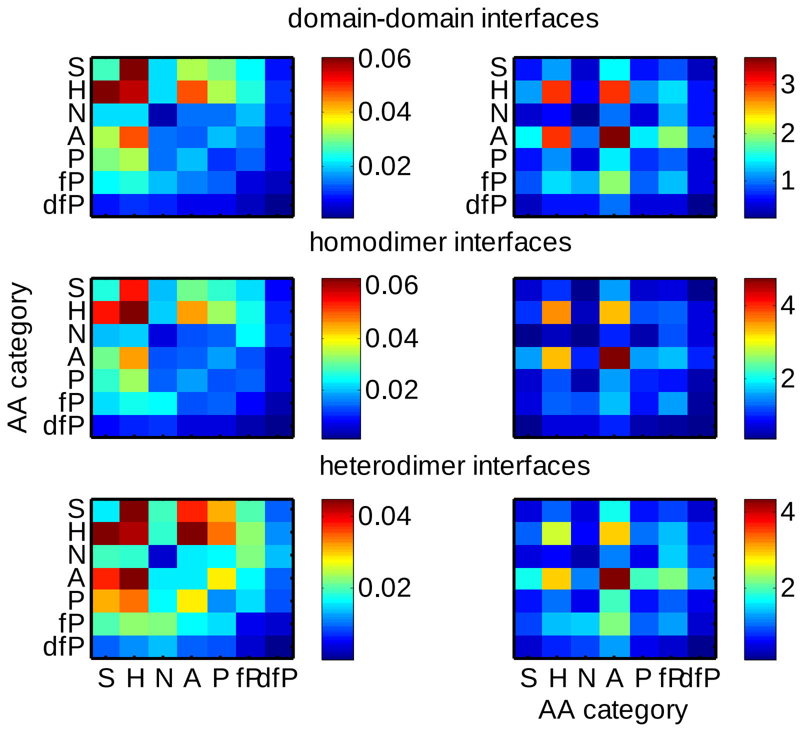

Figure S2. Relative frequencies of contact pairs. The left column presents the observed relative frequencies of different types of contact pairs in the interfaces. The right column shows the ratios between the observed relative frequencies of pair types in the interfaces and their background relative frequencies on the surfaces.

To compare the pattern of the contact pairs among these three data sets of interfaces, we use the same color scale for the observed contact pair types in three data sets in Figure S3.

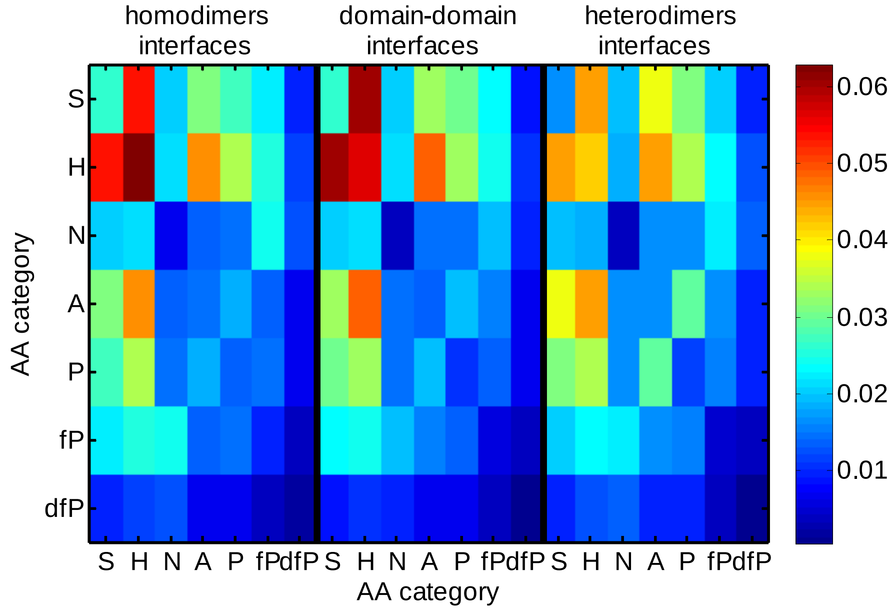

Figure S3. Comparison of the observed relative frequencies of different types of contact pairs among three data sets of interfaces.

From Figure S3, we can see the similar patterns of the contact pairs occurring in the interfaces, but there are also some differences. For example, although S-H and H-H are favored by three kinds of interfaces, S-H is the most frequent contact pair type in the domain-domain interfaces, while H-H is the most frequent one at the interfaces for homo- and hetero- dimmers; the rarest contact pair type is dfP-dfP for all three kinds of interfaces.

Contact triangles

As described in the main text, the observed relative frequencies of the contact triangles can be compared with its background relative frequency on the surface. Figure S4 shows the scatter plots for three data sets. The observed most frequent contact triangle is S-H-H in both the both the domain-domain interfaces and the homodimer interfaces, while S-H-A is the most frequent one in the heterodimer interfaces. In all three data sets, A-A-A is the one with the largest ratio between the observed relative frequency and the background relative frequency, which suggests that it is the most favored contact triangle at interface excluding the confounding effects of the surface.

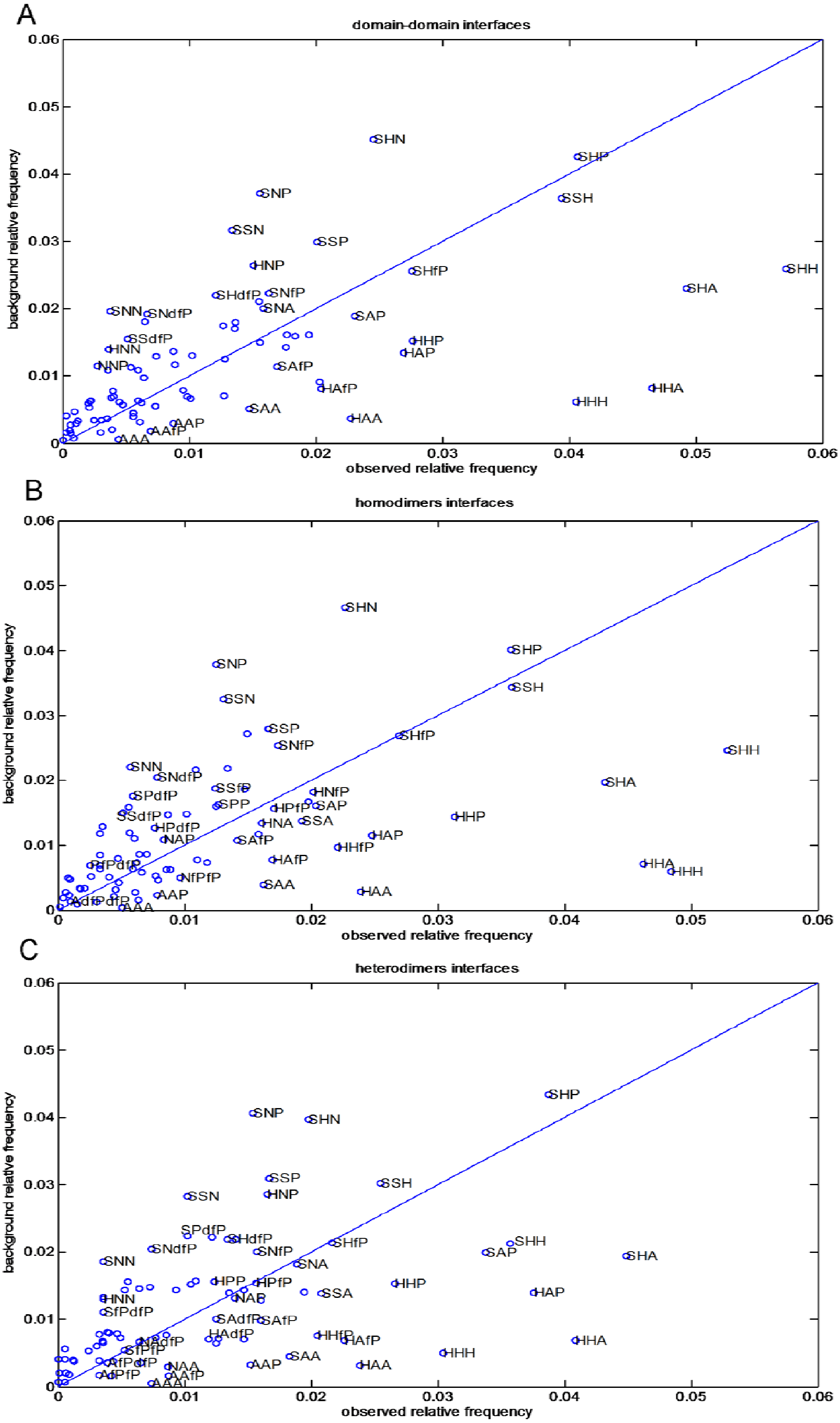

Figure S4. The scatter plots of the observed relative frequencies for the contact triangles against the background relative frequencies in different data sets. A. The domain-domain interfaces; B. The homodimer interfaces; C. The heterodimer interfaces.

Contact 4-tuple

As the supplementary to the main text, the actual numbers of different 4-node-graphs are listed in Table S2.

Table S2. Comparison of the observed frequencies of the contact 4-tuples at interface in three data sets.

	domain-domain interfaces	homodimers interfaces	heterodimers interfaces
A1	531579	287153	33396
A2	4581918	2538900	264243
A3	70082	29941	4410
B1	153568	69301	10545
C1	25195	14914	1677
C2	404	184	18
D1	279400	139679	19004
D2	189657	84109	13510
E1	119930	61360	8513
E2	8389	3372	672
F1	15067	7088	1150

Scoring decoys

In the DOCKGROUND[1], the decoys are listed with there accuracy measurements against the real structure. Table S3 gives an example of the measurements.

Table S3. The accuracy of the decoys in DOCKGROUND.

Decoys	R_rmsd	L_rmsd	I_rmsd	fnat	fnon-nat
r-l_51	0.5300	49.1700	19.7600	0	1
r-l_161170	0.5300	4.7700	2.0800	0.8700	0.1500

NOTE: According to DOCKGROUND, we have the following definitions.

R_rmsd : the RMSD of backbone atoms (N, Ca, C, O) of receptor residues calculated after finding the best superposition of bound and unbound structure.

L_rmsd : the RMSD of the backbone atoms of the ligand after receptor was optimally superimposed.

I_rmsd : the RMSD of the backbone atoms of the interface residues after they have been optimally superimposed.

fnat : the number of native (correct) residue-residue contacts in the predicted complex divided by the number of contacts in the native complex.

fnon-nat: the number of non-native (incorrect) residue-residue contacts in the predicted complex divided by the total number of contacts in that complex.

---

[1] Liu S, Gao Y, Vakser IA (2008) Dockground protein-protein docking decoy set. Bioinformatics 24: 2634-2635.

if l_rmsd < 5 Å, the prediction is called near native.

The local network patterns established in this paper were applied to screening predicted protein-protein interfaces. The chi-square signal was calculated as described in the Method section of the main text for the contact pairs, triangles, and 4-tuples, respectively, and the results are reported in Figure S5. Based on the local network patterns established from the data set of domain-domain interfaces, the chi-square scores are calculated for 28 types of contact pairs (upper graph in Figure S5A), 84 types of contact triangles (middle graph in Figure S5A) and 210 types of contact 4-tuples (lower graph in Figure S5A). Similarly, the chi-square scores based on the data sets of the homodimer interfaces and the heterodimer interfaces are presented in Figure S5B and Figure S5C, respectively. The pair-type signature is not very informative, the triangle-type signature is somewhat informative; it is the 4-tuple signature which most clearly indicates that the decoy r-l_51 deviates from the background, whereas r-l_161170 is a near-native interface.

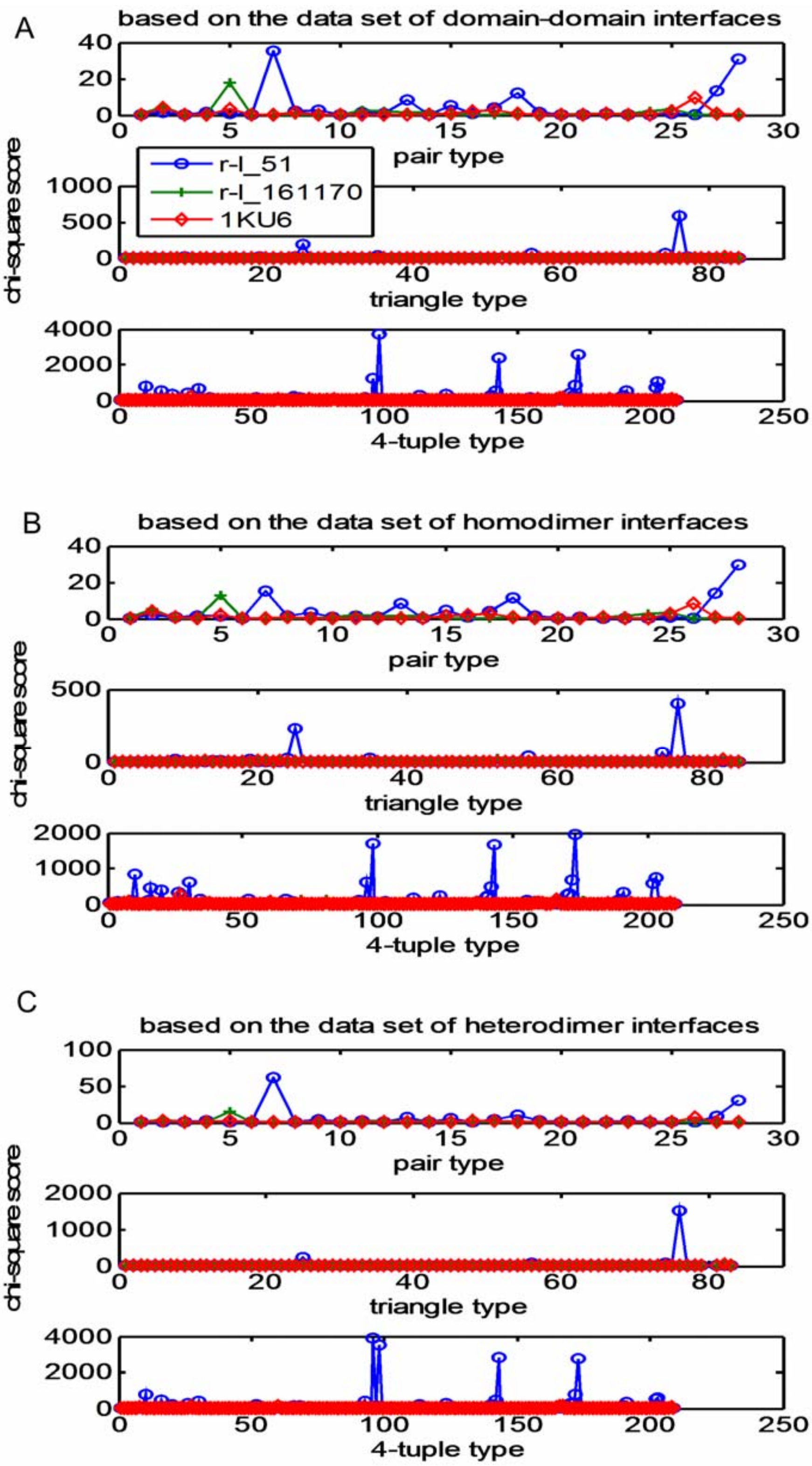

Figure S5. Chi-square scores. The signature established with the chi-square scores calculated by comparing the local network patterns in the predicted interface with the profiles of those patterns revealed in this paper based on three data sets of interfaces. The 4-tuple signature reveals most clearly that the decoy r-l_51 deviates from the background, whereas r-l_161170 is a near-native interface.

Based on the results shown in Figure S5, the following scoring method is established for the docking decoys. If we order the triangle types according to their relative frequency from least to most, we have the following results listed in Table S4. Actually, in the heterodimer interfaces, no 'NNN' and 'dfPdfPdfP' have been observed, while there are 54 'NNN' and 10 'dfPdfPdfP' in the homodimer interfaces, and 42 N-N-N and 7 'dfPdfPdfP' in the domain-domain interfaces.

Table S4. The rarest triangle types at interface.

	r-l_51	r-l_161170	1ku6	Domain-domain	Homo	Hetero
1	'SHA'	'SHfP'	'SHdfP'	'dfPdfPdfP'	'dfPdfPdfP'	'NNN'
2	'SNA'	'SNP'	'SNA'	'fPdfPdfP'	'fPdfPdfP'	'dfPdfPdfP'
3	'SAP'	'SNdfP'	'SNdfP'	'NNN'	'PdfPdfP'	'fPdfPdfP'
4	'SAfP'	'SAfP'	'SAP'	'fPfPdfP'	'NNN'	'SdfPdfP'
5	'SPfP'	'SPfP'	'SPdfP'	'PdfPdfP'	'fPfPdfP'	'PdfPdfP
80	'fPdfPdfP'	'NPP'	'HPP'	'HHH'	'SSH'	'SHH'
81	'SNdfP'	'SHA'	'SPfP'	'SHP'	'SHA'	'HAP'
82	'HNdfP'	'HAfP'	'HAfP'	'HHA'	'HHA'	'SHP'
83	'HPdfP'	'NAP'	'HPfP'	'SHA'	'HHH'	'HHA'
84	'HfPdfP'	'HPP'	'HAP'	'SHH'	'SHH'	'SHA'

Now with the chi-square scores for each type of 4-tuples, we can build a score for a given protein-protein interface.

The scoring results are presented in Figure S6 and Figure 10. The results suggest that the near-native structures as well as the real structure of 1KU6 generally have higher scores (all near-native structures are marked at the upper left corner) than the decoys. While this is a very naïve scoring method, we can still see the good performance of this score. It suggests that the local network patterns at interface revealed in this paper do capture some main features of the protein-protein interfaces. To combine the local network pattern counts with more information from other sources would be a promising future research direction of this work.

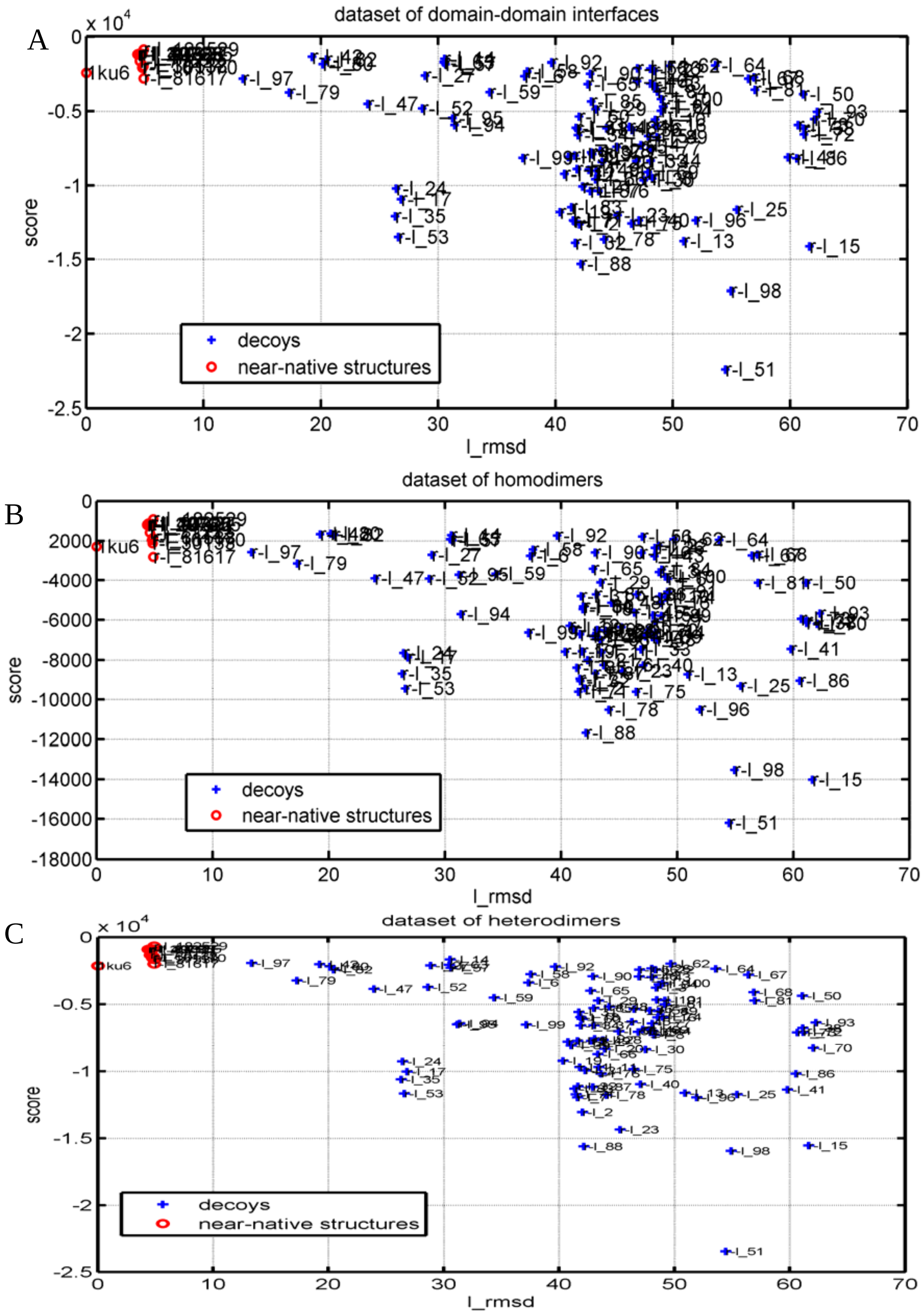

Figure S6. Scores against l_rmsd. Scores established by the profiles of the local network patterns given by different data sets of interfaces. A. the domain-domain interfaces; B. the homodimer interfaces; C. the heterodimer interfaces.

The scores from iScore (established by the network pattern observed on heterodimers) are plotted against the corresponding l_RMSD’s of all decoys for each protein in the data set as follows (Figure S7). There are about 100 decoys and 1 to 10 near-native structures in each data source, but only those decoys with iScores comparable to the near-native structures are shown in the figures to get a clearer view of the results. Among the 15 complexes in DOCKGROUND which have an interface given by only two chains and 100 decoys and 1-10 near native structures, the lowest iScore was a decoy for all 15 complexes; the highest iScore was a near-native for 10 of the complexes (1e96, 1gpw, 1ma9, 1s6v, 1xd3, 3fap, 1ku6, 1ohp, 1tmq, 1u7f); the top 5 highest iScore's contained at least one near-native structure for 13 of the 15 complexes (1e96, 1gpw, 1ma9, 1s6v, 1xd3, 3fap, 1ku6, 1ohp, 1tmq, 1u7f , 2bkr, 2ckh, 2a5t).

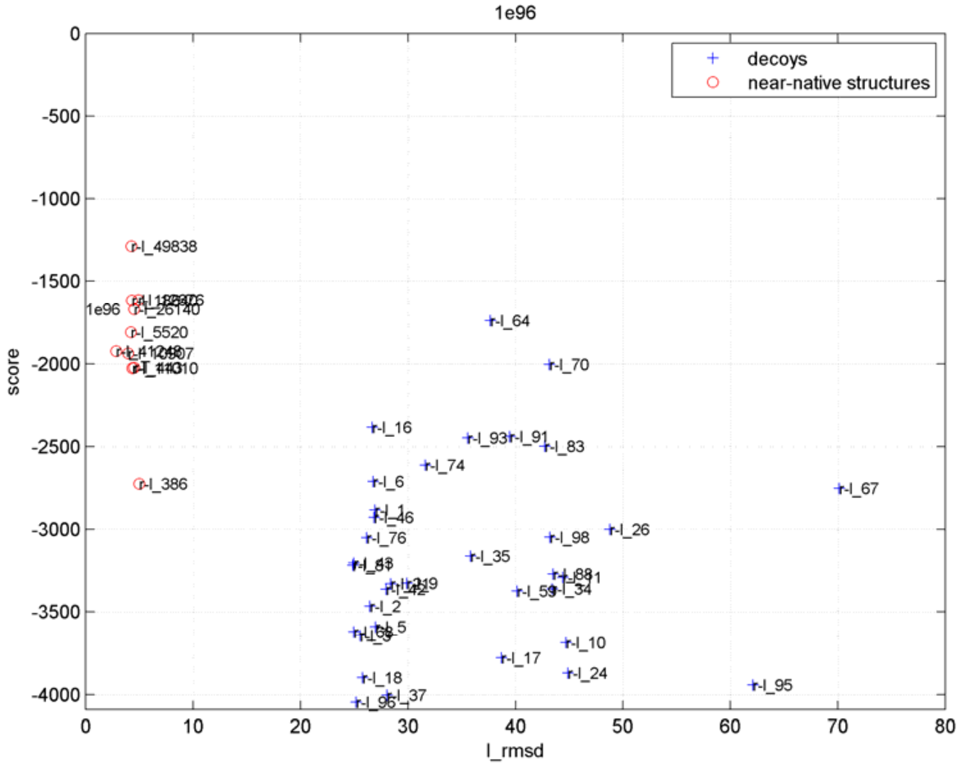

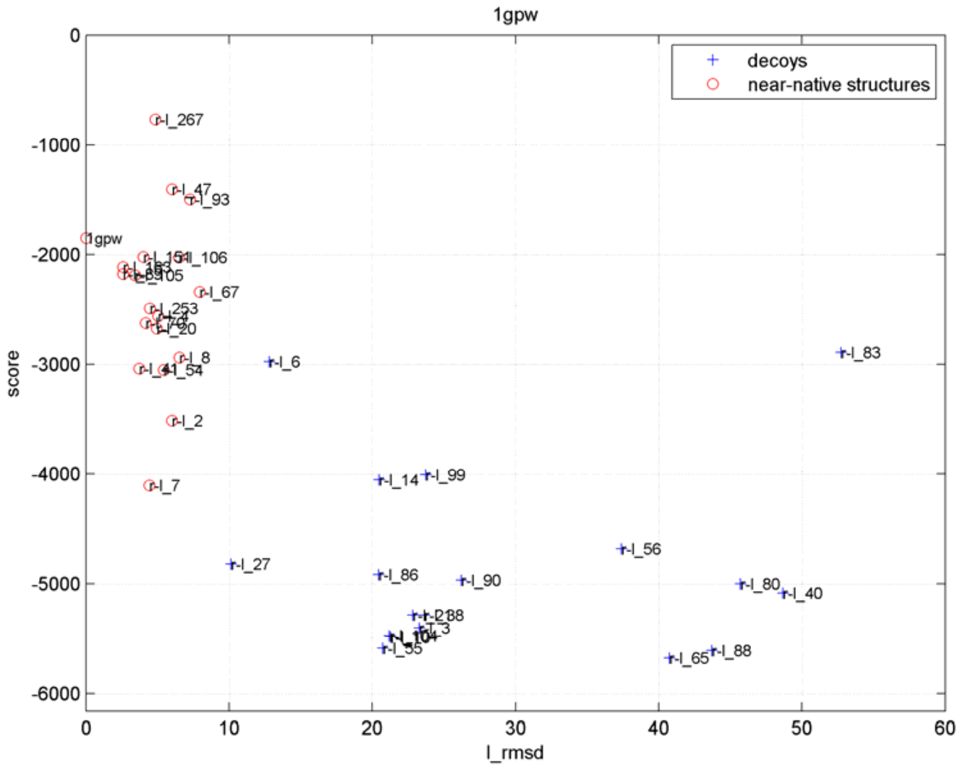
1gpw
decoys
near-native structures
score
l_rmsd
0
-1000
-2000
-3000
-4000
-5000
-6000
0
10
20
30
40
50
60

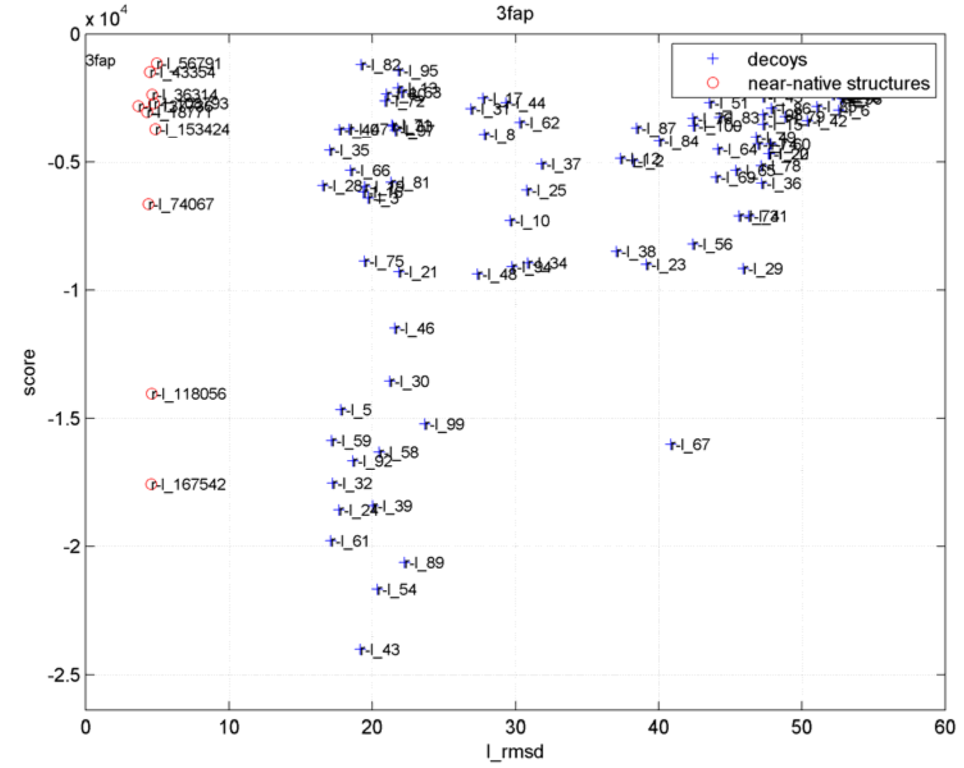
3fap
x 10^4
decoys
near-native structures
score
l_rmsd
0
-0.5
-1
-1.5
-2
-2.5
0
10
20
30
40
50
60

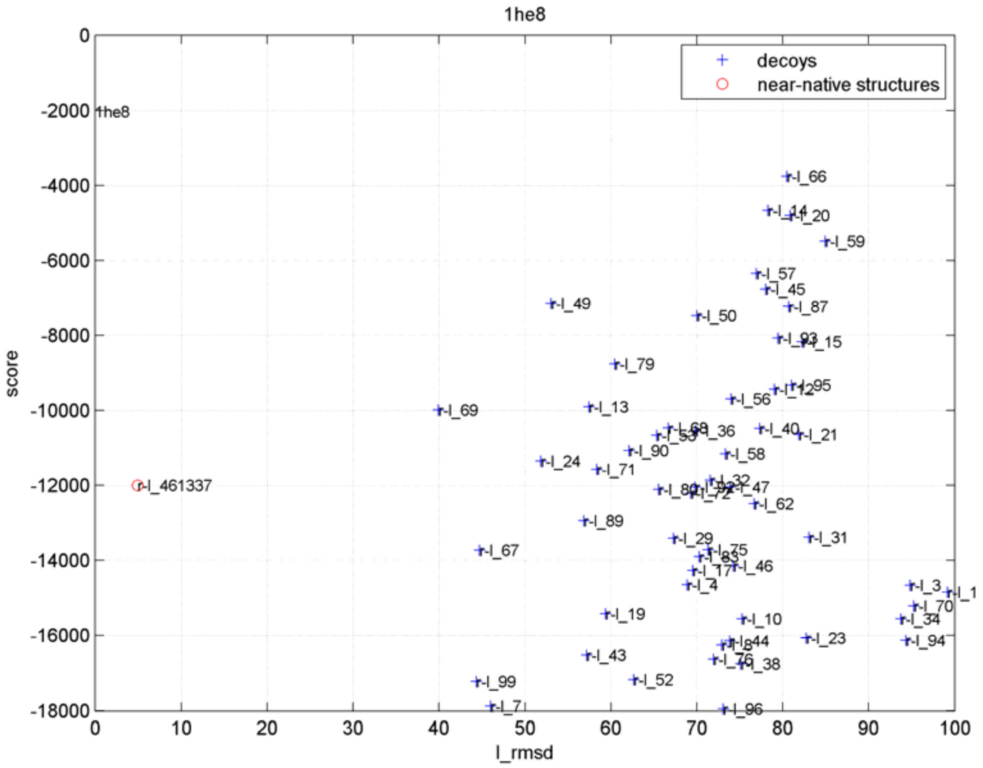
1he8
decoys
near-native structures
score
l_rmsd

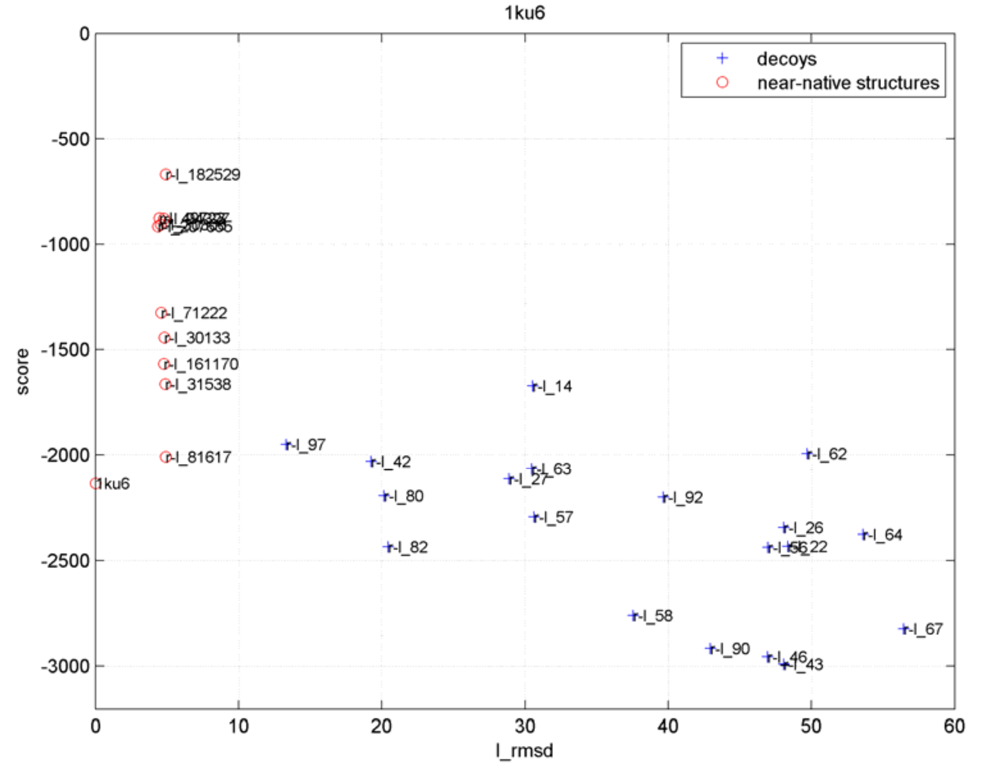
1ku6
decoys
near-native structures
score
l_rmsd

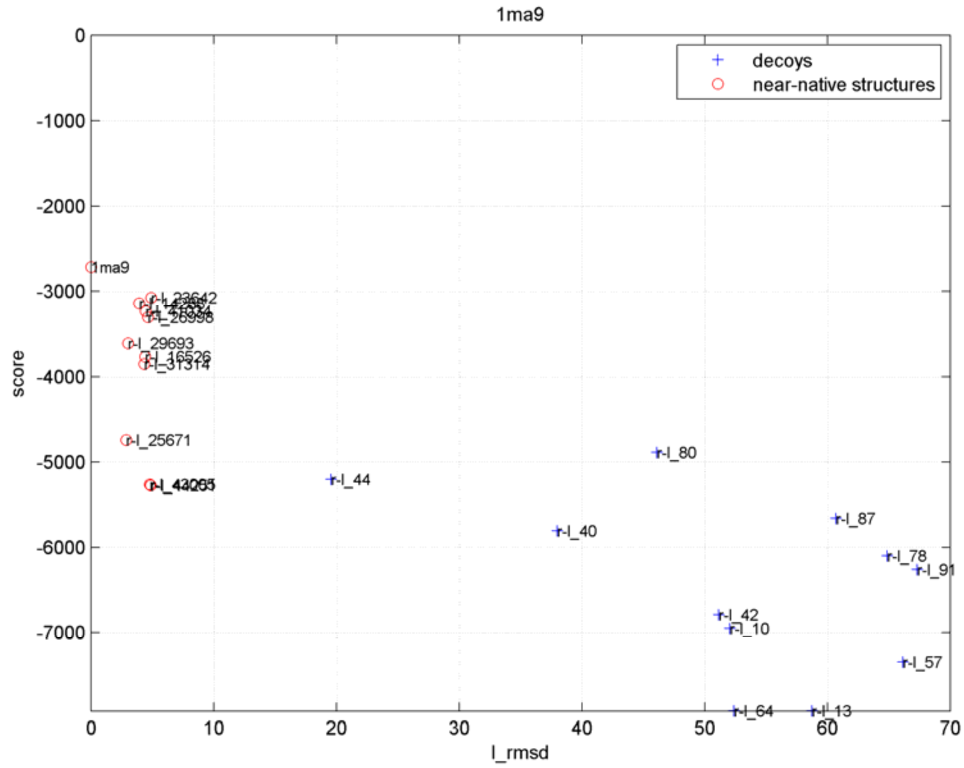
1ma9
decoys
near-native structures
score
l_rmsd
1ma9
r-l_23642
r-l_29693
r-l_16526
r-l_31314
r-l_25671
r-l_44
r-l_80
r-l_40
r-l_87
r-l_78
r-l_91
r-l_42
r-l_10
r-l_57
r-l_64
r-l_13

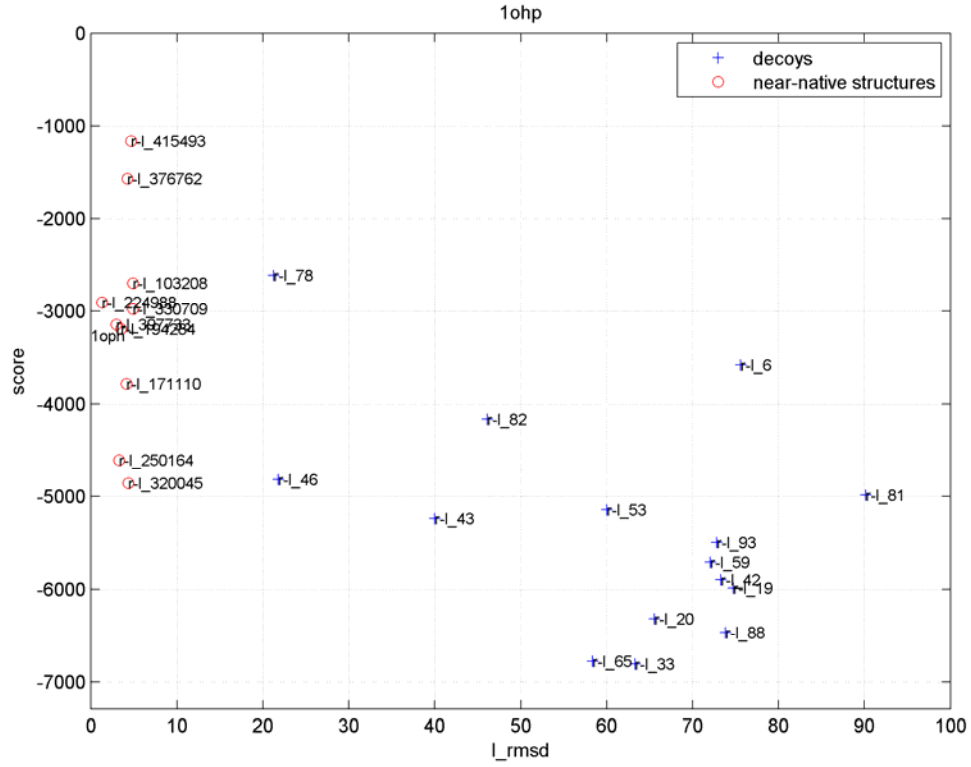
1ohp
decoys
near-native structures
score
l_rmsd
r-l_415493
r-l_376762
r-l_103208
r-l_78
r-l_224988
r-l_330709
r-l_194264
r-l_6
r-l_171110
r-l_82
r-l_250164
r-l_320045
r-l_46
r-l_43
r-l_53
r-l_81
r-l_93
r-l_59
r-l_42
r-l_19
r-l_20
r-l_88
r-l_65
r-l_33

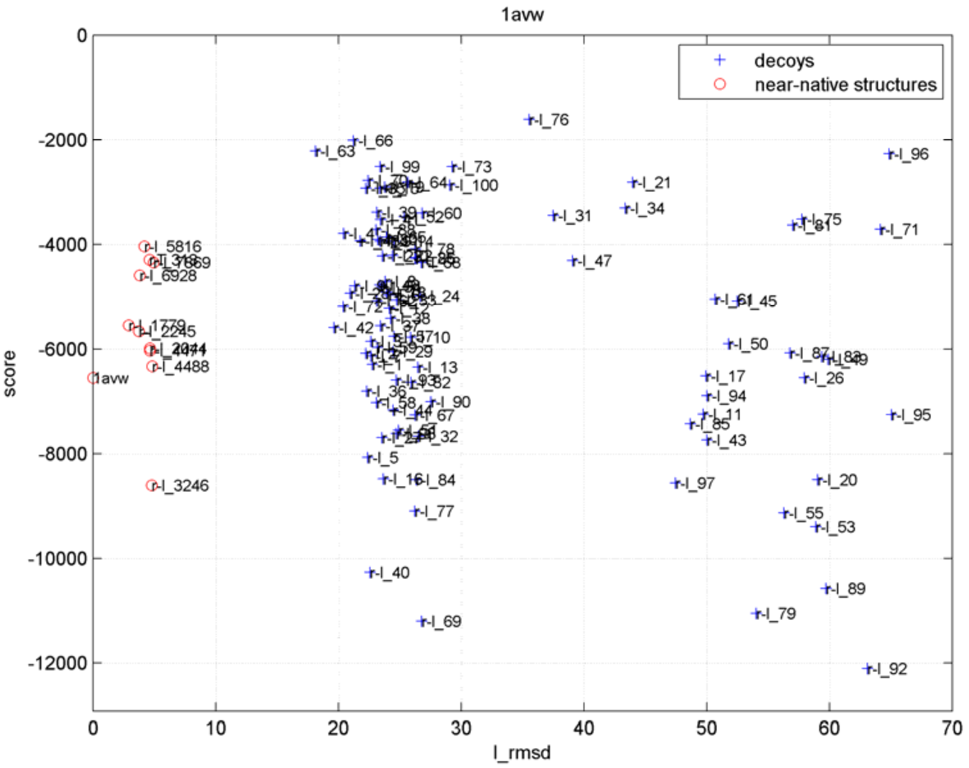
1avw
decoys
near-native structures
score
l_rmsd

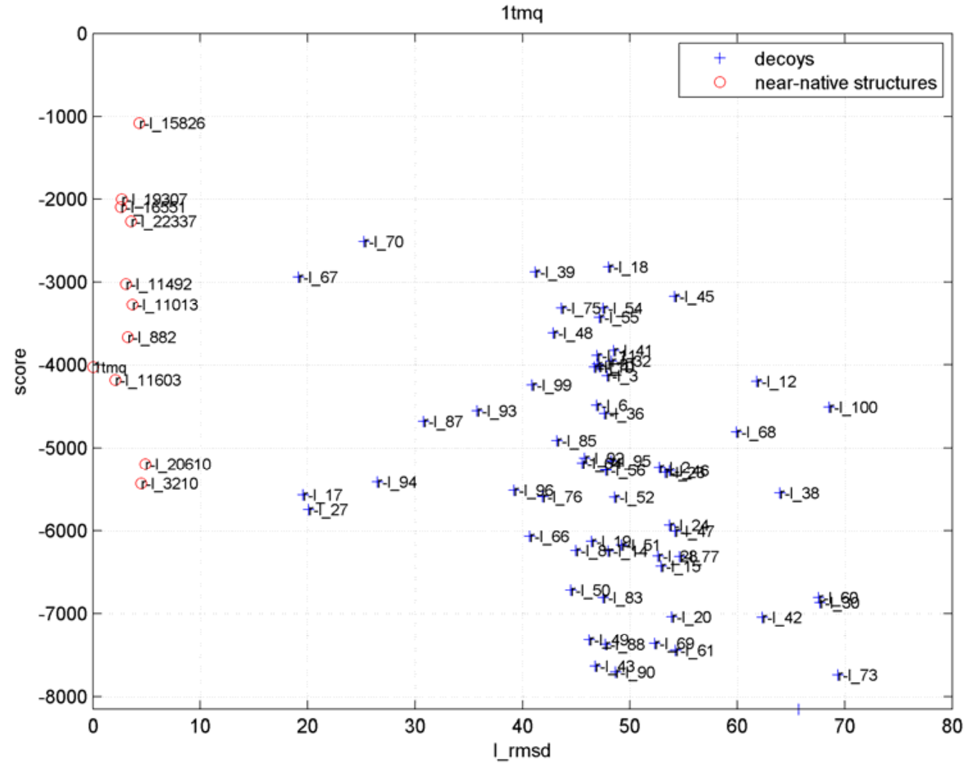
1tmq
decoys
near-native structures
score
l_rmsd

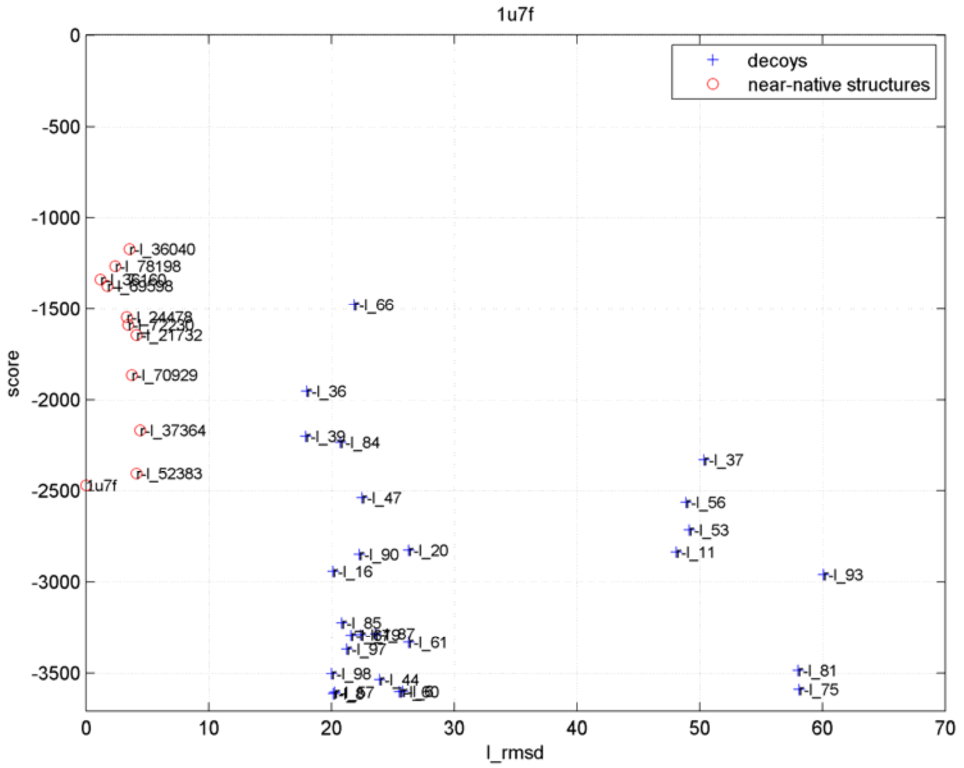
1u7f
decoys
near-native structures
score
l_rmsd
r-l_36040
r-l_78198
r-l_70929
r-l_37364
r-l_52383
1u7f
l_66
l_36
l_39
l_84
l_37
l_47
l_56
l_53
l_11
l_20
l_90
l_16
l_93
l_85
l_61
l_97
l_98
l_44
l_81
l_75

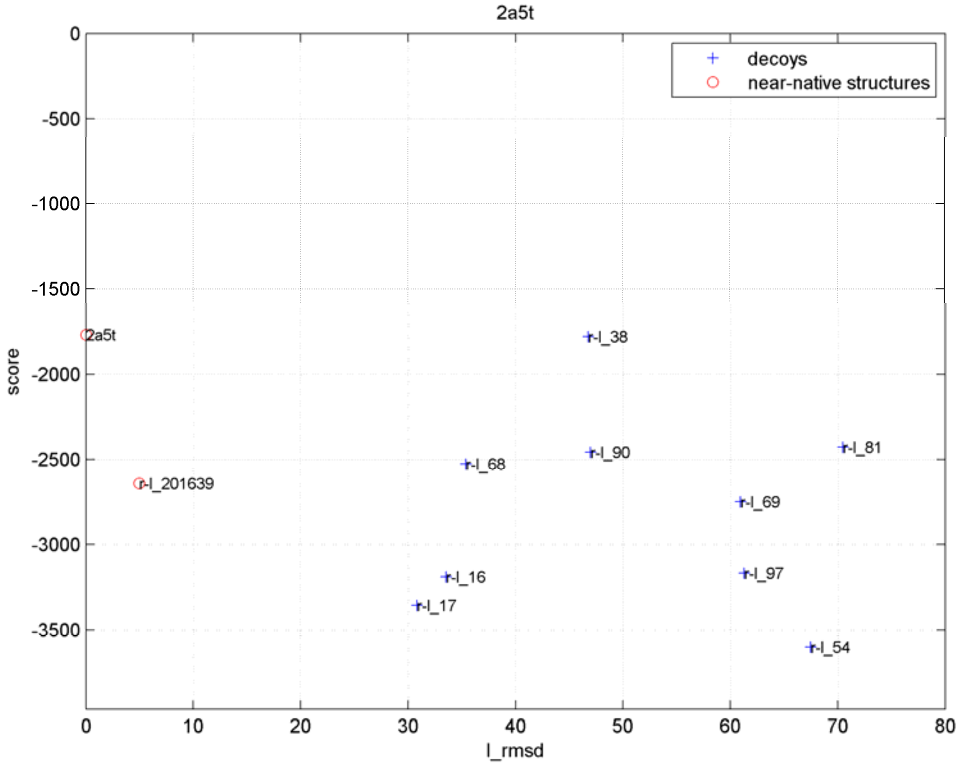
2a5t
decoys
near-native structures
score
l_rmsd
2a5t
r-l_201639
l_38
l_90
l_81
l_68
l_69
l_16
l_97
l_17
l_54

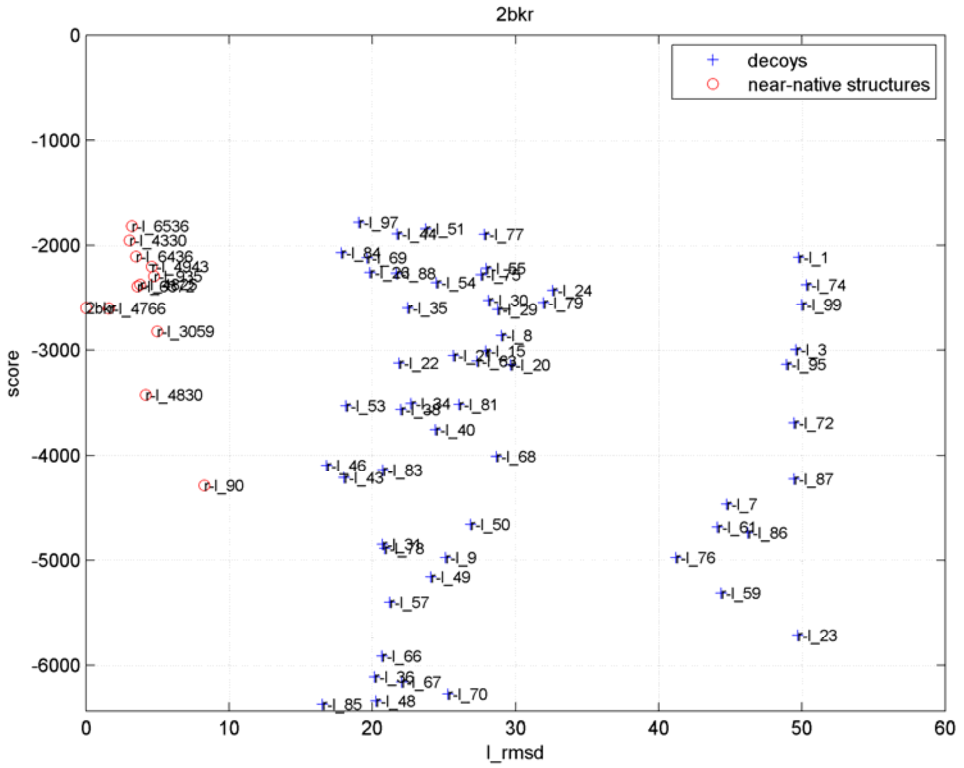
2bkr
score
l_rmsd
0
-1000
-2000
-3000
-4000
-5000
-6000
0
10
20
30
40
50
60
decoys
near-native structures

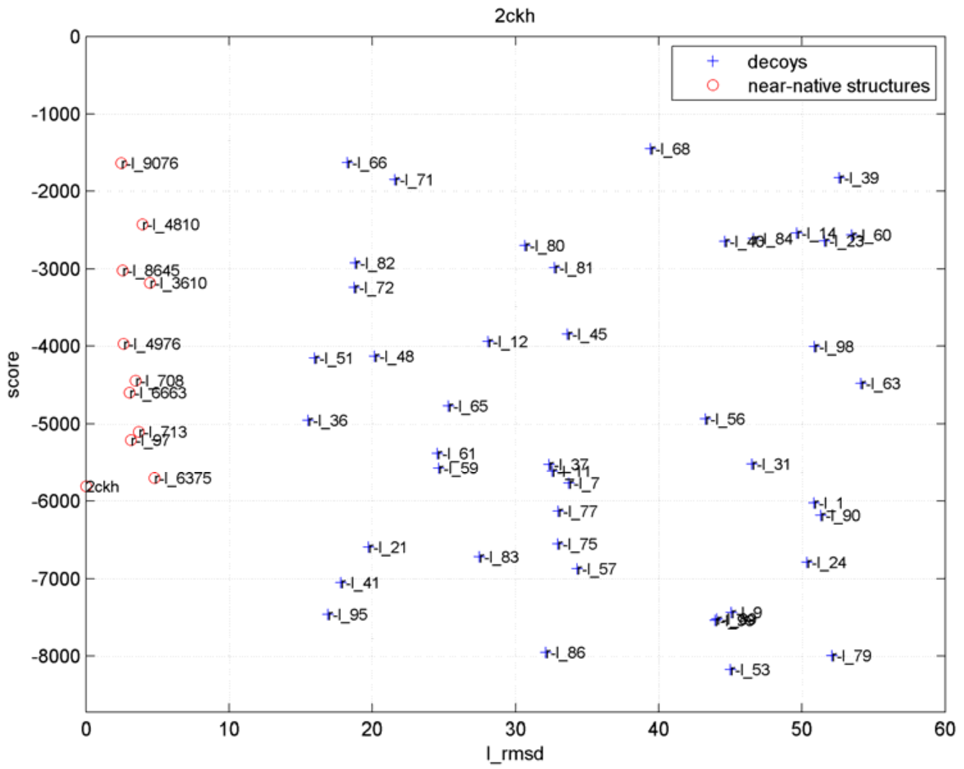
2ckh
score
l_rmsd
0
-1000
-2000
-3000
-4000
-5000
-6000
-7000
-8000
0
10
20
30
40
50
60
decoys
near-native structures

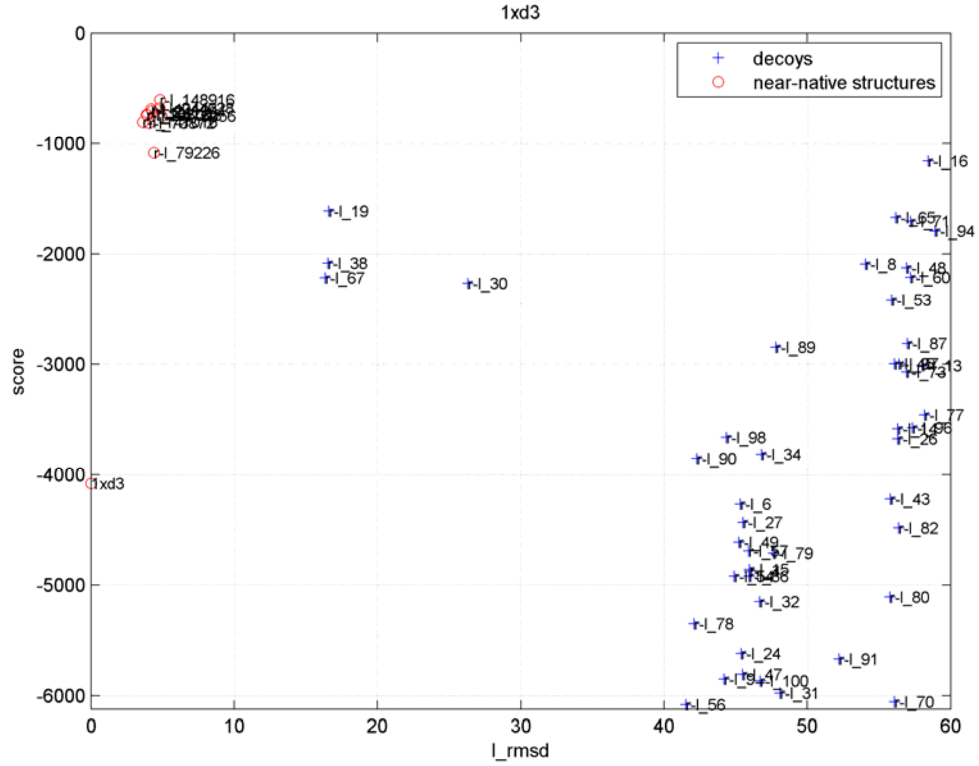

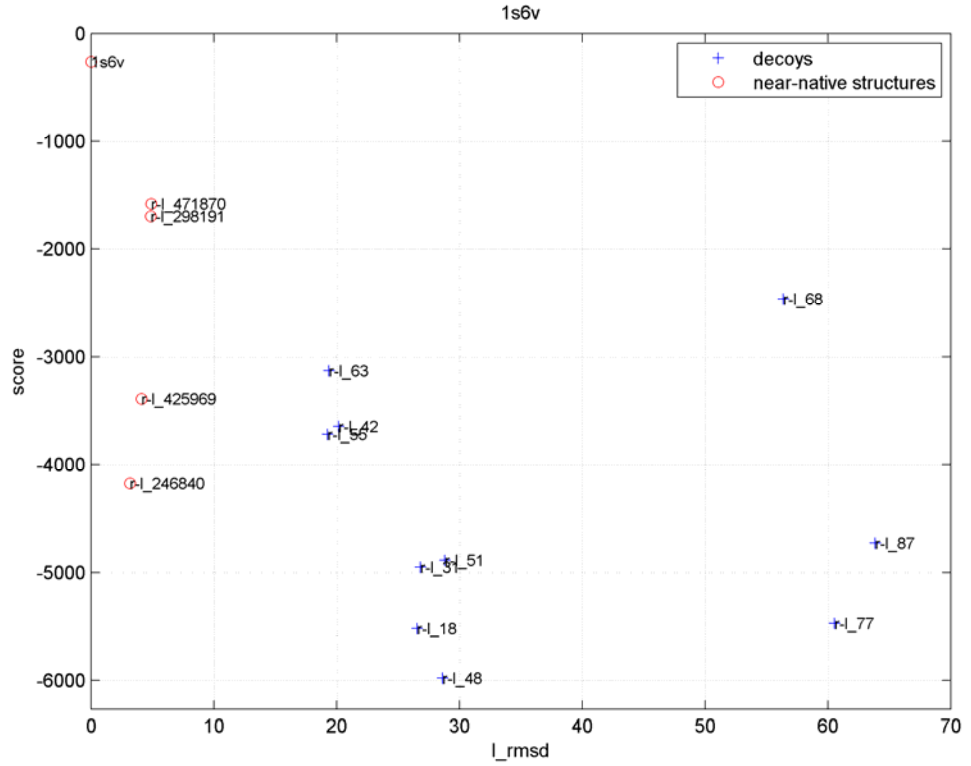

Figure S7. iScore v.s l_rmsd for 15 protein complexes. The name of the protein complex is presented as the title of each graph. There are about 100 decoys and 1 to 10 near-native structures for each complex. To get a clearer view, only those decoys with comparable iScores with the near-native structures are shown. Circles for near-native structures, while plus for decoys.
